# Nonclinical comparability studies of recombinant human arylsulfatase A addressing manufacturing process changes

**DOI:** 10.1371/journal.pone.0195186

**Published:** 2018-04-19

**Authors:** Teresa Wright, Aiqun Li, Jason Lotterhand, Anne-Renee Graham, Yan Huang, Nancy Avila, Jing Pan

**Affiliations:** Shire, Lexington, Massachusetts, United States of America; ITALY

## Abstract

Recombinant human arylsulfatase A (rhASA) is in clinical development for the treatment of patients with metachromatic leukodystrophy (MLD). Manufacturing process changes were introduced to improve robustness and efficiency, resulting in higher levels of mannose-6-phosphate and sialic acid in post-change (process B) compared with pre-change (process A) rhASA. A nonclinical comparability program was conducted to compare process A and process B rhASA. All doses were administered intrathecally. Pharmacodynamic comparability was evaluated in immunotolerant MLD mice, using immunohistochemical staining of lysosomal-associated membrane protein-1 (LAMP-1). Pharmacokinetic comparability was assessed in juvenile cynomolgus monkeys dosed once with 6.0 mg (equivalent to 100 mg/kg of brain weight) process A or process B rhASA. Biodistribution was compared by quantitative whole-body autoradiography in rats. Potential toxicity of process B rhASA was evaluated by repeated rhASA administration at doses of 18.6 mg in juvenile cynomolgus monkeys. The specific activities for process A and process B rhASA were 89 U/mg and 106 U/mg, respectively, which were both well within the target range for the assay. Pharmacodynamic assessments showed no statistically significant differences in LAMP-1 immunohistochemical staining in the spinal cord and in most of the brain areas assessed between process A and B rhASA-dosed mice. LAMP-1 staining was reduced with both process A and B rhASA compared with vehicle, supporting its activity. Concentration–time curves in cerebrospinal fluid and serum of cynomolgus monkeys were similar with process A and B rhASA. Process A and B rhASA were similar in terms of their pharmacokinetic parameters and biodistribution data. No process B rhASA-related toxicity was detected. In conclusion, manufacturing process changes did not affect the pharmacodynamic, pharmacokinetic or safety profiles of process B rhASA relative to process A rhASA.

## Introduction

Metachromatic leukodystrophy (MLD) is a rare autosomal recessive lipid metabolism disorder that leads to neurological and cognitive decline in affected individuals. The disorder is caused by a deficiency of active lysosomal enzyme arylsulfatase A (ASA), resulting in accumulation of sulfatide in glial cells and some neurons in the central nervous system (CNS) and the periphery [1, 2]. Three clinical subtypes of MLD are recognized according to the age of onset: late-infantile (symptom onset ≤3 years of age); juvenile (4–16 years of age) and adult (≥16 years of age) [2]. Individuals homozygous for a null allele develop the most severe, late-infantile form that is associated with rapid motor function decline and typically results in death during childhood [2, 3].

With no approved treatments for the underlying disease, management of patients with MLD typically consists of general measures to alleviate symptoms and the provision of supportive care [4]. Hematopoietic stem cell transplantation has been used for several decades and has shown some benefits in patients with MLD, but only when performed in pre-symptomatic individuals with juvenile or adult MLD [3, 5]. Gene therapies, administered via hematopoietic stem cells or directly into the brain, have shown some activity in animal models of MLD [6, 7], and are in early-stage clinical development [8]. An ongoing phase 1/2 trial of lentiviral vector-mediated hematopoietic stem cell gene therapy in patients with late-infantile or early-juvenile MLD has shown promising preliminary results [9]. At a minimum of 18 months of follow-up, disease onset was prevented or disease progression halted in eight patients, all but one of whom were treated when they were pre-symptomatic [9]. Enzyme replacement therapy has so far been the most successful approved therapy for patients with other lysosomal storage diseases, including Gaucher disease, Fabry disease and Hunter syndrome [10, 11]; however, applicability to MLD has, until recently, been hampered by the limited ability of ASA to penetrate the blood–brain barrier to treat the CNS manifestations of the disease [12].

Intrathecal drug administration has been used to deliver large biomolecules, such as ASA, to the brain successfully [12–15]. Furthermore, because some of these biomolecules transfer from the cerebrospinal fluid (CSF) to blood, there is some drug penetration into the periphery, beyond the site of administration [12]. Recombinant human ASA (rhASA, HGT-1110), which has the same structure as endogenous human ASA, is in clinical development for the treatment of patients with late-infantile MLD, with all current studies using a surgically placed intrathecal drug delivery device. A phase 1/2, multicenter, dose-escalation study of enzyme replacement therapy with rhASA in children with late-infantile MLD was started in 2012 and recently completed (ClincialTrials.gov identifier: NCT01510028) [16, 17], with a long-term extension initiated in 2013 (ClincialTrials.gov identifier: NCT01887938) [18]. The original clinical trial application was supported by a nonclinical program similar to that described by Pfeifer *et al*. [13], and in line with good laboratory practice for nonclinical laboratory studies [19].

In preparation for pivotal phase 3 trials, changes were introduced to the manufacturing process of rhASA to improve the robustness and efficiency of the manufacturing technique. These manufacturing process changes resulted in increased levels of sialic acid and mannose-6-phosphate (M6P) in the post-change (process B) product compared with pre-change (process A) product, as well as a slight increase in purity. Sialic acid is important in determining uptake into the liver by the asialoglycoprotein receptor [20, 21], and M6P is critical for uptake by the M6P receptor into target cells [22, 23]. Comparability studies were conducted because of the potential for these differences to lead to increased tissue uptake of, and serum exposure to, process B rhASA.

Here we describe an evaluation of the activity, safety and pharmacokinetics of process B rhASA compared with process A rhASA in a nonclinical comparability program (Table 1), in accordance with the International Conference on Harmonisation (ICH) Q5E guidance [24].

**Table 1:** Nonclinical studies assessing the potential biological implications of difference in critical quality attributes between process B and process A rhASA.

Quality attributes of process B vs process A rhASA	Potential biological implications	Nonclinical endpoints	Nonclinical studies
Increased purity	No difference in toxicity expected	Toxicity	11-week, repeated-dose toxicology study in juvenile cynomolgus monkeys
Increased M6P levels	Higher cell/tissue uptake	Toxicity	11-week, repeated-dose toxicology study in juvenile cynomolgus monkeys
		Biodistribution	Rat QWBA with tissue AUC
		Activity	Multiple endpoint pharmacodynamics study in immunotolerant MLD mice
Increased sialic acid levels	Higher serum exposure; minimal impact expected on CSF clearance	Serum and CSF pharmacokinetics	Pharmacokinetic crossover study in juvenile cynomolgus monkeys

AUC, area under the concentration–time curve; CSF, cerebrospinal fluid; M6P, mannose-6-phosphate; MLD, metachromatic leukodystrophy; QWBA, quantitative whole-body autoradiography; rhASA, recombinant human arylsulfatase A.

## Materials and methods

### Materials

Four batches of rhASA were manufactured using process B in compliance with Good Manufacturing Practices [25]. From these four batches, the lot that exhibited the highest levels of M6P and sialic acids and the highest mean relative potency by cell uptake bioassay was chosen for use in nonclinical comparability studies because it was considered to have the greatest potential differences relative to process A rhASA. The clinical lot chosen from the process A batches was selected based on availability and expiration date. The hydrolytic activity of the chosen process A and process B clinical lots was analyzed using the artificial substrate *para*-nitrocatechol sulfate (Sigma).

### Pharmacodynamic comparability

Pharmacodynamic comparability of rhASA manufactured using process A and process B was assessed in immunotolerant MLD mice aged 4–5 months at study initiation. Methodology similar to that described in previous reports evaluating the activity of rhASA in mice was used [22, 26]. The assessment was carried out at Shire (Lexington, MA, USA); the facilities at Shire are accredited by the Association for Assessment and Accreditation of Laboratory Animal Care (AAALAC) International. All experiments were approved by the Shire Institutional Animal Care and Use Committee (IACUC) (protocol number MLD.01.14) and conformed to the Guide for the Care and Use of Laboratory Animals published by the US National Institutes of Health (NIH Publication No. 85–23, revised 1996). All mice were provided food and water *ad libitum*.

Immunotolerant MLD mice are ASA knockouts (ASA−/−) that have a stably integrated transgene coding for an inactive variant of human ASA, making them immunologically tolerant to injected rhASA [22, 26]; these were provided courtesy of Volkmar Gieselmann and Ulrich Matzner (University of Bonn, Bonn, Germany). Animals were assigned to one of six groups: untreated (n = 6); vehicle (154 mM NaCl, 0.005% polysorbate 20, pH 6.0, n = 4); process A rhASA at 0.04 mg (n = 9) and 0.21 mg (n = 10); process B rhASA at 0.04 mg (n = 10); and 0.21 mg (n = 10). Animals were dosed intrathecally on days 1, 8, 15, and 21 or 22. A group of seven untreated C57/B16 mice served as wild-type controls. Animals were sacrificed 24 hours after their final dose of rhASA.

Histological analysis involved immunohistochemical staining of lysosomal-associated membrane protein-1 (LAMP-1), a lysosomal protein marker used for the detection of lysosomal storage diseases and as an indicator of disease state [27]. LAMP-1 immunostaining of mouse brain and spinal cord was performed in 5 μm paraffin sections using rabbit polyclonal anti- LAMP-1 antibody (Abcam, Cat # ab24170, Lot # ER127402-2, 1:400, Cambridge, MA, USA) and Bond™ Polymer Refine Detection Kit (Leica, Cat# DS9800, Buffalo Grove, IL, USA). LAMP-1 signal was obtained by staining with 3, 3-diaminobenzidine and followed by counterstaining with hematoxylin. The primary antibody was replaced by isotype IgG for the negative control. Digital images of LAMP-1-stained slides were created and analyzed using an Aperio scanner with ImageScope software (Leica Microsystems Inc., Buffalo Grove, IL, USA). The relative staining positivity was obtained using the following equation: positivity (%) = (LAMP-1-positive pixel number/total pixel number) × 100. Statistical comparison of the vehicle and untreated animals was performed for each tissue using a Mann–Whitney test. Based on this evaluation (p > 0.01 between groups for all tissues), data were combined into a single group (hereafter referred to as control). Data were normalized to the control group and statistical comparisons were performed using a one-way analysis of variance with Tukey's multiple comparison test. Comparisons with *p* < 0.01 were deemed statistically significant.

### Pharmacokinetic comparability

Pharmacokinetic comparability of rhASA manufactured using process A and process B was assessed in a two-phase crossover study in juvenile (2–3 years of age) cynomolgus monkeys. The study was conducted at Northern Biomedical Research in Spring Lake, MI, USA, which is an AAALAC International-accredited facility. The study complied with the Final Rules of the US Animal Welfare Act Regulations (Title 9, Code of Federal Regulations) and the US Guide for the Care and Use of Laboratory Animals (Institute for Laboratory Animal Research, National Research Council; 8th edition) (protocol number 047–041). The study was also conducted in accordance with recommendations made in the Weatherall report, The use of non-human primates in research [28]. Animals were housed individually in stainless steel cages. Room temperature was maintained at 23 ± 5°C, with relative humidity at 50 ± 20% and a minimum of 10 air-changes per hour. A 12-h light-dark cycle was used, which could be interrupted as required to ascertain the health of the animal. The animals were provided with appropriate enrichment including food, water, treats, vitamin supplements, and environmental enrichment such as toys, swings, perches and mirrors. Every effort was made to minimize pain, discomfort and suffering by using appropriate methods and agents for analgesia, anesthesia and euthanasia as described below. All animals were under the care and supervision of veterinary staff.

In phase 1, animals were randomized based on body weight to either process A rhASA 6.0 mg (n = 6 [3 male, 3 female]) or process B rhASA 6.0 mg (n = 6 [3 male, 3 female]) groups. An intrathecal lumbar catheter was implanted surgically following pre-treatment with subcutaneous atropine sulfate (0.01 mg/kg), followed by anesthesia under intramuscular ketamine hydrochloride (8 mg/kg) and approximately 1 L/min of oxygen and 2% isoflurane. Doses of rhASA were administered via the implanted intrathecal lumbar catheter at a dose volume of 1 mL. The 6.0 mg dose was equivalent to 100 mg/kg of brain weight when normalized to a brain weight of 60 g in the cynomolgus monkey. This dose was based on the highest dose of rhASA that was administered in the phase 1/2 clinical study in children with late-infantile MLD [17, 29, 30]. In that clinical study, the highest dose administered was 100 mg, equivalent to 100 mg/kg of brain weight when normalized to a brain weight of 1 kg (determined for the age group of the study participants [31]).

In phase 2, which started after a washout period of at least 8 days, animals were dosed with process B rhASA if they had been dosed with process A rhASA in phase 1 and, conversely, animals were dosed with process A rhASA if they had been dosed with process B rhASA in phase 1. The animals were humanely euthanized up to 24 hours after the last dose of rhASA.

Body weights, food consumption and clinical parameters were monitored, and blood and CSF samples were collected pre-dose and at pre-specified intervals post-dose. Quantitation of the levels of rhASA in the serum and CSF of juvenile cynomolgus monkeys was achieved using GLP-validated ELISA assay methods (validated at Covance Laboratories, Chantilly, VA, USA). Microtiter plates were coated with a rabbit-derived anti-rhASA polyclonal antibody (SH040). After incubation with samples and standards, the microtiter plates were washed and horseradish peroxidase-conjugated anti-ASA mouse monoclonal antibody (clone 19-16-3) was added to all wells. The lower limit of quantification for monkey serum was 39.1 ng/mL and for CSF was 19.5 ng/mL, and the upper limit of quantification for each method was 1250 ng/mL.

### Biodistribution comparability

Tissue distribution, disposition kinetics and excretion profiles of rhASA manufactured using process A and process B were assessed in male Sprague Dawley^®^ rats, conducted at WIL Research (Ashland, OH, USA). The facilities at WIL Research are accredited by AAALAC International and all experiments were approved by the WIL Research IACUC (protocol number WIL-771087). The study was conducted in compliance with the USA Food and Drug Administration Good Laboratory Practice Regulations [19]. Rats were housed individually with free access to food and water.

The study used quantitative whole-body autoradiography (QWBA) and gamma counting after a single intrathecal dose of [^125^I]-rhASA. To assess tissue distribution, 28 rats implanted with intrathecal ports were randomized so that each received a dose of [^125^I]-rhASA 0.62 mg manufactured using either process A (14 animals) or process B (14 animals). Two rats from each dose group were euthanized at each of seven time points after dosing (approximately 1, 4, 12, 24, 48, 96 and 168 hours post-dose) and were processed for QWBA, with radioconcentration determined for selected tissues. Blood samples were collected from isoflurane anesthetized animals via cardiac puncture before euthanasia. To assess pharmacokinetic parameters, aliquots of whole blood, plasma and tissue were analyzed for total radioactivity using gamma counting. To assess rhASA excretion patterns, six male Sprague Dawley rats were assigned to a group receiving [^125^I]-rhASA 0.62 mg manufactured using either process A (3 animals) or process B (3 animals). Rats were housed in individual metabolism cages for separate collection of urine and feces during the 168 hours following administration of [^125^I]-rhASA.

### Toxicology

The toxicology profile of repeated intrathecal administration of rhASA manufactured using process B was assessed in juvenile (aged <1 year at initiation) cynomolgus monkeys at Covance Laboratories, Münster, Germany. The study facility was accredited by AAALAC International. All study procedures complied with the German Animal Welfare Act and were approved by the local IACUC (protocol number 8297167). The study adhered to the Commission of the European Communities Recommendation 2007/526/EC on guidelines for the accommodation and care of animals used for experimental and other scientific purposes and was in line with the USA Food and Drug Administration Good Laboratory Practice Regulations [19]. Animal welfare standards were similar to those described above for the pharmacokinetic comparability studies.

An intrathecal port catheter was implanted surgically following pre-treatment with meloxicam (0.2 mg/kg), followed by ketamine hydrochloride (5 mg/kg) and medetomidine (0.06 mg/kg). Doses of rhASA were administered by slow-bolus delivery using the intrathecal port catheter system. Animals were dosed every 2 weeks for 11 weeks (six doses in total) with either process B rhASA 18.6 mg (n = 8 [4 male, 4 female]) or vehicle control (NaCl 154 mM, 0.005% polysorbate 20, pH 6.0; n = 8 [4 male, 4 female]) at a dose volume of 0.6 mL. The maximum feasible dose of rhASA of 18.6 mg was used in this study and was based on the highest tolerable volume in the juvenile cynomolgus monkey (0.6 mL) and on the concentration of the process B rhASA formulation (31.0 mg/mL).

Assessments included clinical examinations (body weight; food consumption; physical, cardiovascular [electrocardiogram and blood pressure], neurological and ophthalmological evaluations) and pathological examinations (serum chemistry; hematology; coagulation; urinalysis; CSF cell count and CSF chemistry). Antigenicity of process B rhASA was assessed in serum and CSF. Physical and neurological examinations were performed on unsedated animals twice during the pre-dose phase (before surgery and ≥5 days after surgery), and once during weeks 1, 5 and 9 of the dosing phase (approximately 24 hours after dosing) as well as once before necropsy. Electrocardiography investigations were performed on non-anesthetized, temporarily restrained animals during the pre-dose phase (after surgery) and in week 11 of the dosing phase (close to dosing and 3 hours post-dose). All animals were humanely euthanized 24 hours after the final dose. Complete sets of tissues were collected, but only the target tissues identified in the original study with process A (i.e. spinal cord and dorsal root ganglia) were subjected to histopathological evaluation.

## Results

### Pharmacodynamic comparability

The specific activities for process A and process B rhASA were 89 U/mg and 106 U/mg, respectively, which were both well within the target range for the assay. The comparison of the two materials indicated that the greatest changes were in the M6P and sialic acid contents, and the specific activities were similar.

**Fig 1:**
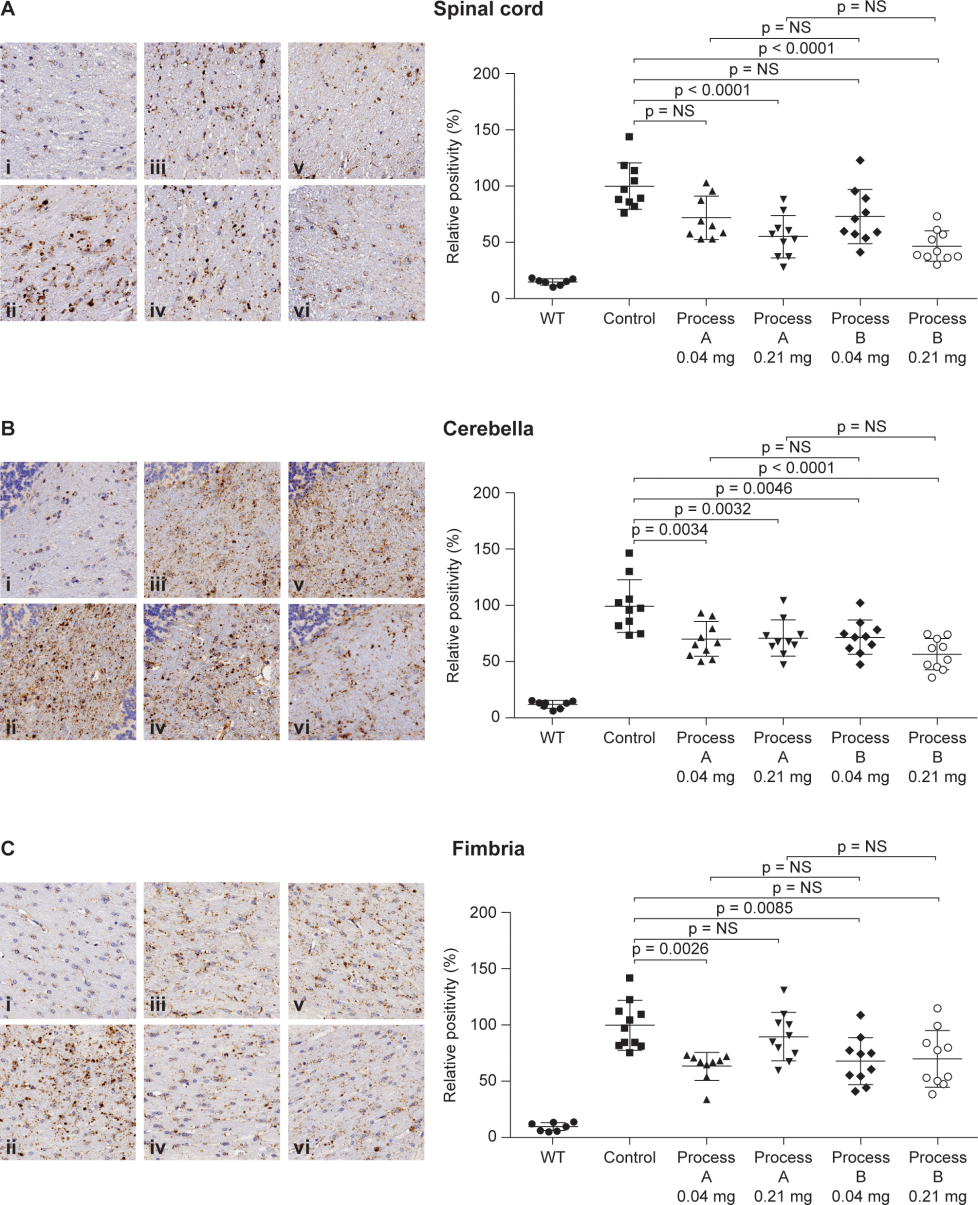
LAMP-1 staining in MLD mice treated with process A or process B rhASA. Representative images of immunohistochemical staining of LAMP-1 with corresponding morphometric analysis in the white matter of (A) spinal cord, (B) cerebella and (C) fimbria of immunotolerant MLD mice treated with rhASA 0.04 mg or 0.21 mg from process A (iii, v) or process B (iv, vi), or control (ii). Untreated C57/B16 mice served as WT controls (i). Individual and mean values can be found in S1 Table. LAMP-1, lysosomal-associated membrane protein-1; MLD, metachromatic leukodystrophy; NS, not significant; rhASA, recombinant human arylsulfatase A; WT, wild type.

Results of LAMP-1 staining in immunotolerant MLD mice are shown in Fig 1 for the spinal cord, cerebellar white matter and fimbria, and in S1 Fig for the cerebral peduncle, cerebral cortex and striatum. Regions shown to be affected by treatment with process A material from previous studies were chosen for evaluation [22]. No statistically significant differences in LAMP-1 staining in the spinal cord, cerebellar white matter and fimbria were observed between animals dosed with process A versus process B rhASA (Fig 1). Similarly, no statistical differences in staining were observed between process A and process B rhASA in the cerebral peduncle, but differences were observed in the cerebral cortex and striatum (S1 Fig and S2 Table). These differences were considered to be sporadic and not biologically relevant because statistical significance was reached for only one of the two doses tested.

Compared with control animals, there were statistically significant reductions in LAMP-1 staining with both process A and process B rhASA in the spinal cord (0.21 mg dose groups, *p* < 0.0001; Fig 1A), in the cerebellar white matter (0.04 mg and 0.21 mg dose groups, *p* < 0.01; Fig 1A), in the cerebral peduncle (0.04 mg and 0.21 mg dose groups; S1A Fig) and in fimbria (0.04 mg dose groups, *p* < 0.01; Fig 1C). Statistically significant reductions in LAMP-1 staining also occurred with process B rhASA in the cerebral cortex (0.04 mg and 0.21 mg dose groups, *p* ≤ 0.0001; S1B Fig) and in the striatum (0.04 mg dose group, *p* = 0.0005; S1C Fig).

### Pharmacokinetic comparability

**Fig 2:**
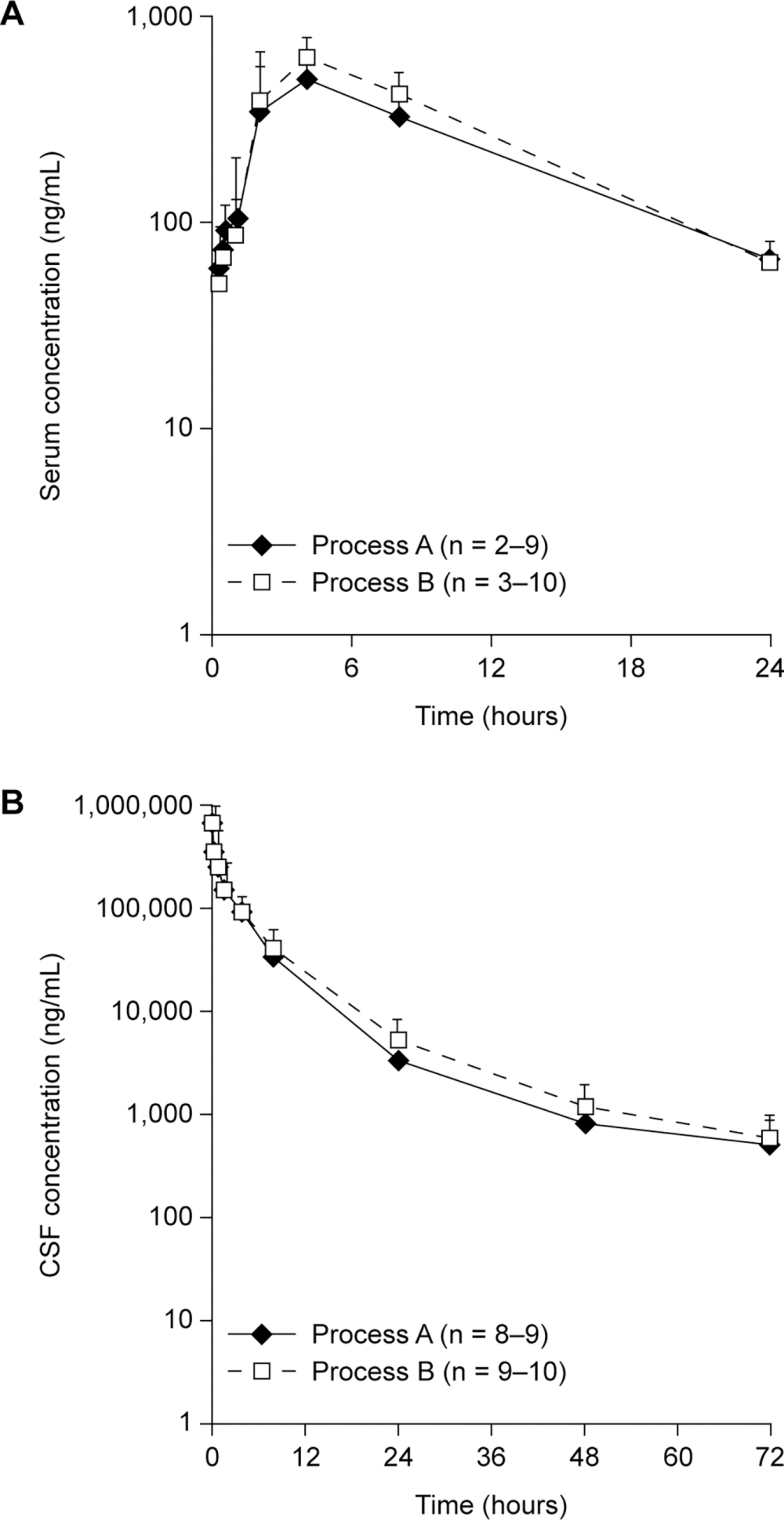
Pharmacokinetic comparability of process A and process B rhASA in juvenile cynomolgus monkeys. Concentration–time curves of process A and process B rhASA in (A) serum and (B) CSF after a single 6.0 mg intrathecal dose in juvenile cynomolgus monkeys. Individual and mean values can be found in S3 and S4 Tables. CSF, cerebrospinal fluid; rhASA, recombinant human arylsulfatase A.

Concentration–time curves of rhASA in CSF and serum after a single 6.0 mg dose in juvenile cynomolgus monkeys were similar when using process A and process B rhASA (Fig 2). Process A and process B rhASA were similar in terms of their pharmacokinetic parameters, including the maximum plasma concentration (C_max_), exposure (area under the concentration–time curve from time 0 to the last measurement [AUC_last_]) and clearance (Table 2).

**Table 2:** Mean cerebrospinal fluid and serum pharmacokinetic parameters in juvenile cynomolgus monkeys following intrathecal lumbar administration of rhASA 6.0 mg manufactured using process A or process B.

Pharmacokinetic parameter	Process A			Process B		
	Mean	SD	n	Mean	SD	n
Cerebrospinal fluid						
λz, L/h	0.093	0.047	7	0.131	0.133	8
t_½_, h	10.3	7.9	7	7.8	3.3	8
T_max_, h	0.08	0.00	9	0.12	0.07	10
C_max_, ng/mL	702,000	216,000	9	634,000	224,000	10
AUC_last_, h·ng/mL	1,460,000	340,000	9	1,450,000	391,000	10
AUC_inf_, h·ng/mL	1,500, 000	383, 000	7	1,555,000	389,000	8
Vz, mL	67.8	62.9	7	46.3	23.2	8
CL, mL/h	4.2	1.1	7	4.1	1.2	8
MRT_inf_, h	5.0	1.3	7	5.6	1.9	8
Serum						
T_max_, h	4	2	9	4	1	10
C_max_, ng/mL	558	145	9	647	190	10
AUC_last_, h·ng/mL	5,110	2,080	9	5,520	2,660	10

λz, t_½_, Vz, CL and MRT_inf_ could not be determined in serum samples. Individual and mean values can be found in S5 and S6 Tables. AUC_inf_, area under the concentration–time curve from time 0 to infinity; AUC_last_, area under the concentration–time curve from time 0 to the last measurement; CL, clearance; C_max_, maximum plasma concentration; λz, terminal rate constant; h, hour; MRT_inf_, mean residence time to infinity; n, number; t_½_, terminal elimination phase half-life; rhASA, recombinant human arylsulfatase A; SD, standard deviation; T_max_, time to maximum plasma concentration; Vz, volume of distribution.

### Biodistribution comparability

**Fig 3:**
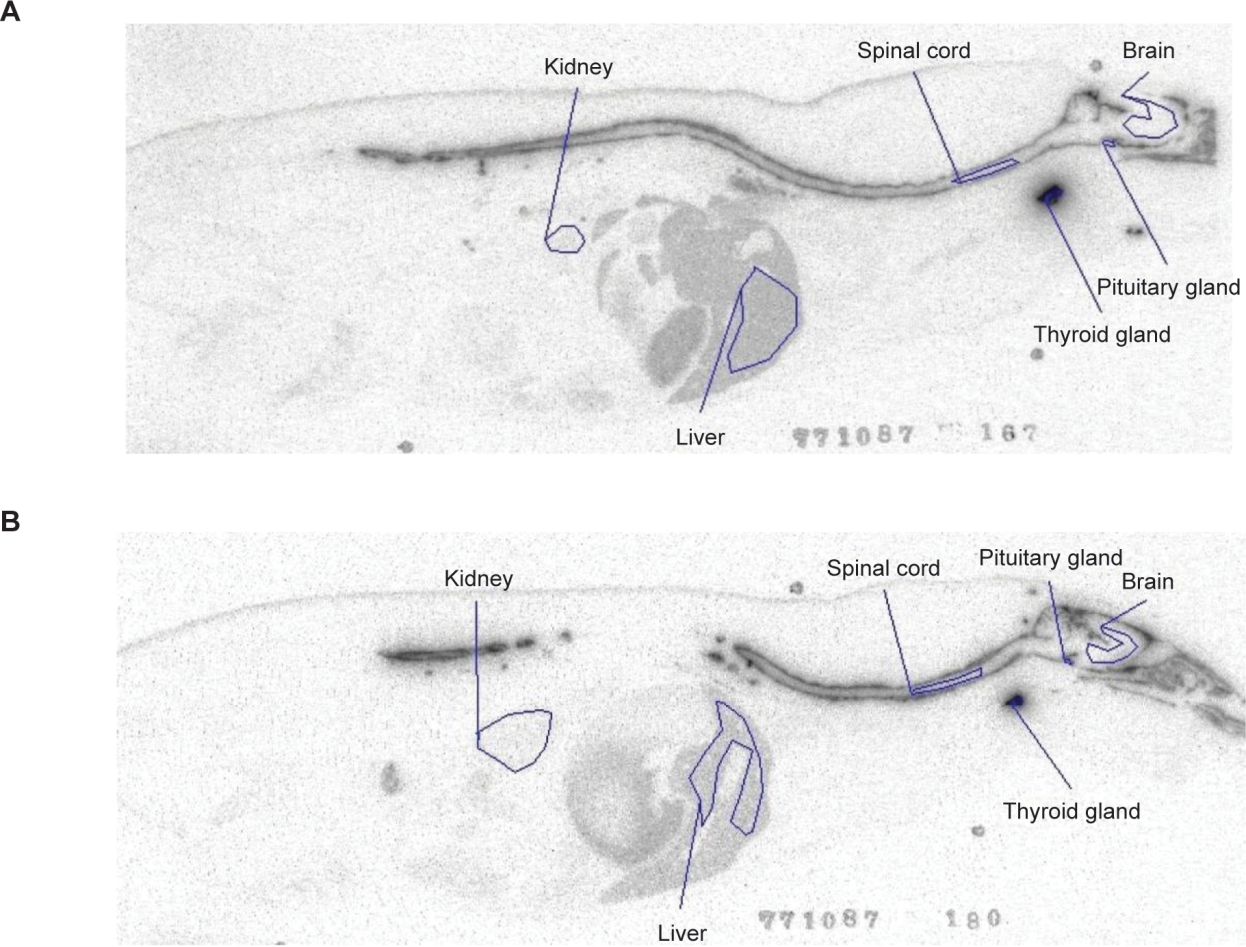
Biodistribution of process A and process B rhASA in rats. Representative whole body autoradioluminograms showing tissue distribution of radioactivity 4 hours after a single intrathecal dose of (A) process A and (B) process B [^125^I]-rhASA 0.62 mg in male Sprague Dawley rats. rhASA, recombinant human arylsulfatase A.

Trichloroacetic acid precipitation of plasma samples indicated that approximately 33% of ^125^I was unbound to rhASA. Fig 3 depicts representative whole body autoradioluminograms showing distribution of process A and process B [^125^I]-rhASA in male Sprague Dawley rats 4 hours after administration of a single intrathecal 0.62 mg dose. C_max_ and AUC_last_ for process A and process B rhASA, and the ratios of process B rhASA to process A rhASA in different tissues are shown in Table 3. The highest calculated concentration (C_max_) of [^125^I]-rhASA was found in the thyroid, likely due to the uptake of unbound iodine. This was followed by the pituitary, spinal cord and liver, which all had similar levels (Table 3). The lowest concentrations of [^125^I]-rhASA occurred in fat, testes, muscle and eyes (Table 3). When inspected visually (Fig 3), the apparent concentration in the spinal cord was very low relative to the concentration in the neighboring CSF and meninges, suggesting that the calculated values in the spinal cord may have been overestimated. The close proximity to regions with a high concentration of ^125^I and small quantitation area may have contributed to the overestimation of spinal cord values.

**Table 3:** Pharmacokinetic parameters of rhASA equivalents following a single intrathecal 0.62 mg dose of process A or process B [^125^I]-rhASA in male Sprague Dawley rats.

Tissue	C_max_, ng Eq/g			AUC_last_, ng Eq·h/g		
	Process A	Process B	Ratio B to A	Process A	Process B	Ratio B to A
Plasma	1,219	1,471	**1.21**	27,600	35,200	**1.28**
Whole blood	989	1,038	**1.05**	21,300	25,900	**1.22**
Adrenal gland	2,484	965	**0.39**	37,200	81,600	**2.19**
Bone marrow (femur)	1,261	1,036	**0.82**	84,900	81,100	**0.96**
Bone (femur)	449	594	**1.32**	43,900	36,300	**0.83**
Brain	1,439	2,310	**1.61**	93,800	135,000	**1.44**
Eye	342	349	**1.02**	20,800	12,400	**0.60**
Fat	223	186	**0.83**	13,000	7890	**0.61**
Harderian gland	404	383	**0.95**	31,800	18,300	**0.58**
Heart	495	547	**1.11**	10,500	10,600	**1.01**
Kidney	3,690	867	**0.23**	36,500	53,700	**1.47**
Kidney (cortex)	2,586	914	**0.35**	42,400	60,700	**1.43**
Kidney (medulla)	3,610	905	**0.25**	31,500	46,900	**1.49**
Large intestine	779	780	**1.00**	27,400	33,200	**1.21**
Liver	4,452	4,484	**1.01**	226,000	325,000	**1.44**
Lung	654	1,005	**1.54**	32,900	26,400	**0.80**
Muscle (femoral)	290	344	**1.19**	24,800	17,100	**0.69**
Pancreas	873	561	**0.64**	17,000	13,400	**0.79**
Pituitary gland	6,869	6,689	**0.97**	348,000	527,000	**1.51**
Prostate	650	534	**0.82**	16,300	19,800	**1.21**
Skin	672	588	**0.88**	26,000	59,100	**2.27**
Small intestine	1,107	858	**0.78**	18,200	25,500	**1.40**
Spinal cord	5,955	8,933	**1.50**	452,000	344,000	**0.76**
Spleen	1,625	1,501	**0.92**	70,300	91,200	**1.30**
Stomach	2,258	1,291	**0.57**	43,600	63,700	**1.46**
Testis	272	278	**1.02**	18,600	10,200	**0.55**
Thymus	492	426	**0.87**	7,900	15,000	**1.90**

AUC_last_, area under the concentration–time curve from time 0 to the last measurement; C_max_, maximum plasma concentration; rhASA, recombinant human arylsulfatase A.

C_max_ values for process B rhASA were approximately 1.5-fold higher than those of process A [^125^I]-rhASA for brain, spinal cord and lung (highest ratios), and approximately 0.2–0.6-fold lower than those of process A [^125^I]-rhASA for kidney, adrenal gland, stomach and pancreas (lowest ratios; Table 3). Differences in exposure (AUC_last_) were less than two-fold for all tissues except skin and adrenal gland, which had ratios of 2.27 and 2.19, respectively, for process B to process A [^125^I]-rhASA (Table 3). The terminal elimination phase half-life in plasma was 37.2 hours with process A and 42.5 hours with process B [^125^I]-rhASA.

**Fig 4:**
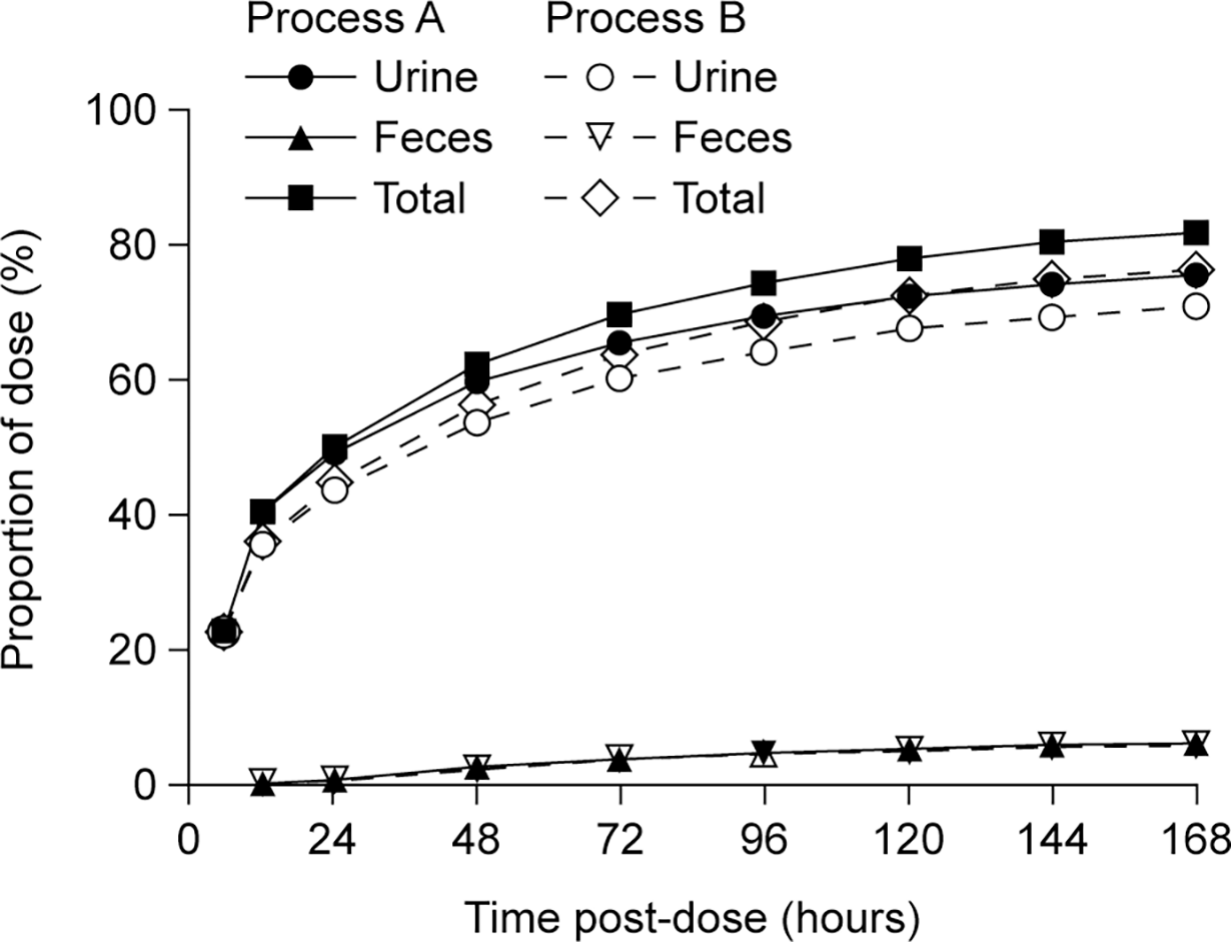
Excretion of process A and process B rhASA in rats. Urinary, fecal and total excretion profiles of process A and process B [^125^I]-rhASA 0.62 mg after a single intrathecal dose in male Sprague Dawley rats (n = 2 and n = 3, respectively). Process A [^125^I]-rhASA 0.62 mg was assigned to three rats, but data from one rat were excluded because the level of radioactivity recovered was less than 1% of what was expected, indicating that this rat had been incorrectly dosed. Individual and mean values can be found in S7 Table. rhASA, recombinant human arylsulfatase A.

Excretion profiles in urine and feces are shown in Fig 4. Excretion patterns were similar for both process A and process B [^125^I]-rhASA. Most process A and process B product was excreted in the urine (76% and 71%, respectively), indicating that rhASA is systemically cleared primarily via renal elimination, and about 6% was excreted in the feces for both products. The proportion of the administered dose recovered in the carcass was 8% for process A product and 6% for process B product, with the majority located in the thyroid, CSF and meninges. The total recovery (all excreta and carcass combined) was 90% and 83% for animals dosed with process A or process B [^125^I]-rhASA, respectively.

### Toxicology

The intrathecal administration of process B rhASA was well tolerated by juvenile cynomolgus monkeys, with no drug-related effects on clinical, neurological or pathological examinations.

The intrathecal administration of process B rhASA at 18.6 mg every 2 weeks for 11 weeks to juvenile cynomolgus monkeys (six doses in total) was the no observable adverse effect level. Administration of process B rhASA was associated with infiltrates with eosinophils in the following regions: meningeal areas around the brain and spinal cord; perivascular areas in the brain and spinal cord; pericanalicular (adjacent to the central canal) areas in the spinal cord; and perineurium/epineurium areas around the spinal nerve roots. None of these process B rhASA-related changes were of a severity that would reasonably be expected to alter the function of the nervous system and, based on morphology alone, none of these process B rhASA-related changes were considered to be adverse effects. The presence of the infiltrates in the spinal cord was consistent with what would be expected in an animal with a protein administered to the intrathecal space [32].

Mean concentration of process B rhASA was 544 ng/mL in serum and 2185 ng/mL in CSF on day 2; at week 11, it was 36 ng/mL in serum and 171 ng/mL in CSF (S8 Table). All rhASA-dosed animals developed anti-drug antibodies by week 10 with higher anti-drug antibody levels observed in serum than in the CSF (mean: 391,461 ng/mL vs 7,382 ng/mL, respectively; S9 Table); anti-drug antibodies did not affect rhASA exposure to target tissues as confirmed by immunohistochemistry and tissue ELISA (data not shown).

## Discussion

The manufacturing process of rhASA was changed to improve process robustness and efficiency preparation for pivotal clinical studies and potential commercialization. A series of comprehensive studies has therefore been conducted to compare the analytical and nonclinical characteristics of rhASA produced using these two manufacturing processes. The strategy for the comparability program was driven by the nature of drug development for rare diseases and the intrathecal route of administration of rhASA. MLD, a rare disease with an incidence of about 1 per 100,000 live births, is categorized into three subtypes based on the age of onset: late-infantile (symptom onset ≤3 years of age); juvenile (4–16 years of age) and adult (≥16 years of age) [2]. The phase 1/2 clinical studies of rhASA enrolled patients with late-infantile MLD who developed symptoms before 30 months of age [16–18]. This late-infantile MLD is the most severe form of the disease and progresses rapidly, with a 5-year mortality of about 50% [3]. In our current studies evaluating process change comparability, the duration and dose levels were based on previously conducted toxicology and pharmacology studies.

The results of the nonclinical comparability studies demonstrate that the pharmacodynamics, pharmacokinetics (in serum and CSF), biodistribution and toxicity are similar for process A (pre-change) and process B (post-change) rhASA. The differences in the critical quality attributes (increased sialic acid and M6P content) resulting from the change to the manufacturing process did not affect the hydrolytic activity of the enzyme or the pharmacodynamic, pharmacokinetic or safety profiles of rhASA.

In addition to demonstrating the comparability of process A and B, the data from our studies also provide evidence for the potential in vivo activity of rhASA, as illustrated by the dose-dependent reductions in LAMP-1 staining of spinal cord and brain, including the white matter and grey matter, in immunotolerant MLD mice. In individuals with lysosomal storage diseases, accumulation of non-metabolized compounds results in an increase in the number and size of lysosomes, and thus an increase in lysosomal proteins, with LAMP-1 levels increasing by up to five-fold [27]. The decrease in LAMP-1 staining observed in the pharmacodynamic comparability study thus serves as a potential indicator of the therapeutic effects of rhASA. Animal studies conducted by other researchers have also yielded safety and activity data for intrathecal drug delivery to the brain, providing further support for this method of administration [12, 13, 15, 32].

The changes in the manufacturing process of rhASA were introduced to improve the robustness and efficiency of the process to support the progression of the clinical study program in MLD. Process A rhASA (produced using the initial manufacturing process) was administered in the initial nonclinical studies, and in the clinical phase 1/2 study and its extension [16, 18]. Based on the results of the nonclinical comparability program, process B rhASA was first used in the phase 1/2 extension study in July 2015 [18]. It is expected that all participants will have transitioned to process B rhASA by the first quarter of 2016.

The analytical differences associated with process B rhASA (higher levels of M6P and of sialic acid) compared with process A rhASA are critical quality attributes of rhASA. The comprehensive comparability studies were performed in accordance with the ICH Q5E guidance [24] and assessed rhASA manufactured using process A and process B in terms of quality, activity and safety. Methods used to establish comparability depend on the type of changes introduced by the manufacturing process modifications. If the changes are minor, straightforward analytical studies may suffice; however, when analytical differences are more substantial, as was the case with rhASA, a comprehensive nonclinical study program is required.

Relevant studies were conducted to evaluate the potential biological effects of increased levels of M6P and sialic acid. These included pharmacodynamic (activity in immunotolerant MLD mice), pharmacokinetic (crossover study in juvenile cynomolgus monkeys), biodistribution (QWBA in the rat) and repeat-dose toxicity (juvenile cynomolgus monkeys) studies. In accordance with ICH QE5 [24], the nonclinical studies performed are considered appropriate to evaluate the nature and extent of the quality attributes affected by process B.

It was considered possible that the higher M6P levels in process B rhASA could lead to increased uptake of rhASA into target tissues via the M6P receptor [33], which could influence efficacy, biodistribution and, potentially, toxicity. Increased rhASA uptake into target tissues (directly or via systemic clearance) could also translate into a greater pharmacodynamic effect as well as higher rhASA exposure in tissues. The data from the nonclinical comparability studies reported here show that these potential effects did not occur with intrathecal dosing of process B rhASA.

The potential biological impact of changes in sialic acid can be assessed only *in vivo*. rhASA is likely to be cleared through the asialoglycoprotein receptor of the liver. Thus, increased levels of sialic acid in process B rhASA could result in increased rhASA serum exposure, due to reduced uptake into the liver. This, however, would not be expected to affect the clearance of rhASA from the CSF because clearance after intrathecal lumbar dosing is similar to the CSF turnover rate, suggesting that CSF bulk flow is the primary mechanism for clearance [34].

Taking into consideration the posology of rhASA and the distribution in serum and CSF following intrathecal administration [35], a study to evaluate pharmacokinetics in both compartments was considered necessary to assess comparability. These parameters were evaluated in the pharmacokinetic crossover study following intrathecal administration of rhASA in juvenile cynomolgus monkeys. Results showed similar rhASA concentration–time curves in CSF and serum after a single 6 mg dose of process A or process B rhASA. The biodistribution study showed some differences between tissues in terms of C_max_ and a slight difference in recovery rate following administration of process A or process B rhASA. These variations might be due to relative binding differences caused by higher levels of M6P and sialic acid in process B than in process A rhASA.

The potential toxicological effect of the increased levels of M6P and sialic acid in process B rhASA on the safety profile of rhASA was evaluated in an 11-week, repeat-dose toxicity study in juvenile monkeys. No unique process B rhASA-related effects were detected. Administration of process B rhASA in monkeys was associated with infiltrates in some regions of the brain and spinal cord. These infiltrates are consistent with findings from other studies where human proteins are administered in the intrathecal space in cynomolgus monkeys [13, 32]. The presence of these infiltrates is a characteristic reaction to administration of a human protein (rhASA) to a non-human primate and provides additional confirmation of exposure to rhASA. None of the rhASA-related changes were deemed to be sufficient to alter the function of the nervous system and thus were considered non-adverse.

## Conclusion

In conclusion, following changes to the manufacturing process of rhASA that resulted in analytical differences between process A and B rhASA, the results from these comprehensive, nonclinical comparability studies show that the differences do not affect the pharmacology, pharmacokinetic, biodistribution or safety profiles of rhASA in animals. The results of these studies endorse the use of process B rhASA in future clinical trials without dose adjustment.

## Supporting information

S1 Fig Additional LAMP-1 staining in MLD mice treated with process A or process B rhASA. Representative images of immunohistochemical staining of LAMP-1 with corresponding morphometric analysis in the (A) cerebral peduncle, (B) cerebral cortex and (C) striatum of immunotolerant MLD mice treated with rhASA 0.04 mg or 0.21 mg from process A (iii, v) or process B (iv, vi), or control (ii). Untreated C57/B16 mice served as WT controls (i). Individual and mean values can be found in S2 Table.
LAMP-1, lysosomal-associated membrane protein-1; MLD, metachromatic leukodystrophy; NS, not significant; rhASA, recombinant human arylsulfatase A; WT, wild type.
(TIF)

S1 Table Morphometry analysis of LAMP-1 staining in white matter of spinal cord and regions of the brain of immunotolerant MLD mice treated with rhASA 0.04 mg or 0.21 mg from process A or process B or control. LAMP-1, lysosomal-associated membrane protein-1; MLD, metachromatic leukodystrophy; rhASA, recombinant human arylsulfatase A; SD, standard deviation; WT, wild-type.
(DOCX)

S2 Table Morphometry analysis of LAMP-1 staining in additional regions of the brain of immunotolerant MLD mice treated with rhASA 0.04 mg or 0.21 mg from process A or process B or control. LAMP-1, lysosomal-associated membrane protein-1; MLD, metachromatic leukodystrophy; rhASA, recombinant human arylsulfatase A; SD, standard deviation; WT, wild-type.
(DOCX)

S3 Table Individual and mean serum concentrations (ng/mL) of rhASA in juvenile cynomolgus monkeys following intrathecal administration of rhASA 6.0 mg manufactured using process A or process B. F, female; h, hour; M, male; rhASA, recombinant human arylsulfatase A. SD, standard deviation.
(DOCX)

S4 Table Individual and mean CSF concentrations (ng/mL) of rhASA in juvenile cynomolgus monkeys following intrathecal administration of rhASA 6.0 mg manufactured using process A or process B. CSF, cerebrospinal fluid; F, female; h, hour; M, male; rhASA, recombinant human arylsulfatase A; SD, standard deviation.
(DOCX)

S5 Table Individual and mean CSF pharmacokinetic parameters in juvenile cynomolgus monkeys following intrathecal lumbar administration of rhASA 6.0 mg manufactured using process A or process B. AUC_inf_, area under the concentration–time curve from time 0 to infinity; AUC_last_, area under the concentration–time curve from time 0 to the last measurement; CL, clearance; C_max_, maximum plasma concentration; CSF, cerebrospinal fluid; F, female; λz, terminal rate constant; h, hour; M, male; MRT_inf_, mean residence time to infinity; n, number; NC, not calculated; t_½_, terminal elimination phase half-life; rhASA, recombinant human arylsulfatase A; SD, standard deviation; T_max_, time to maximum plasma concentration; Vz, volume of distribution.
(DOCX)

S6 Table Individual and mean serum pharmacokinetic parameters in juvenile cynomolgus monkeys following intrathecal lumbar administration of rhASA 6.0 mg manufactured using process A or process B. λz, t_½_, Vz, CL and MRT_inf_ could not be determined in serum samples.
AUC_last_, area under the concentration–time curve from time 0 to the last measurement; h, hour; rhASA, recombinant human arylsulfatase A; SD, standard deviation; T_max_, time to maximum plasma concentration.
(DOCX)

S7 Table Recovery of process A and process B [125I]-rhASA 0.62 mg after a single intrathecal dose in male Sprague Dawley rats. The total excreted in urine was calculated by adding the value for urine, cage rinse and cage wash.
rhASA, recombinant human arylsulfatase A; h, hour.
(DOCX)

S8 Table Individual and mean concentrations (ng/mL) of rhASA in serum and CSF in juvenile cynomolgus monkeys following intrathecal administration of rhASA 18.6 mg manufactured using process B. CSF, cerebrospinal fluid; F, female; M, male; rhASA, recombinant human arylsulfatase A.
(DOCX)

S9 Table Individual and mean concentrations (ng/mL) of anti-rhASA antibodies in serum and CSF in juvenile cynomolgus monkeys following intrathecal administration of rhASA 18.6 mg manufactured using process B. CSF, cerebrospinal fluid; F, female; M, male; rhASA, recombinant human arylsulfatase A.
(DOCX)

## References

[1] Gieselmann V. Metachromatic leukodystrophy: genetics, pathogenesis and therapeutic options. Acta Paediatr Suppl. 2008;97(457):15–21. Epub 2008/05/28. doi: 10.1111/j.1651-2227.2008.00648.xAPA648 [pii]. . 18339182 10.1111/j.1651-2227.2008.00648.x

[2] Gieselmann V, Krageloh-Mann I. Metachromatic leukodystrophy–an update. Neuropediatrics. 2010;41(1):1–6. Epub 2010/06/24. doi: 10.1055/s-0030-1253412 . 20571983 10.1055/s-0030-1253412

[3] Mahmood A, Berry J, Wenger DA, Escolar M, Sobeih M, Raymond G, et al Metachromatic leukodystrophy: a case of triplets with the late infantile variant and a systematic review of the literature. J Child Neurol. 2010;25(5):572–80. Epub 2009/12/30. doi: 10.1177/0883073809341669 ; PubMed Central PMCID: PMC4301611. 20038527 10.1177/0883073809341669 PMC4301611

[4] Batzios SP, Zafeiriou DI. Developing treatment options for metachromatic leukodystrophy. Mol Genet Metab. 2012;105(1):56–63. Epub 2011/11/15. doi: 10.1016/j.ymgme.2011.10.002 . 22078456 10.1016/j.ymgme.2011.10.002

[5] Boucher AA, Miller W, Shanley R, Ziegler R, Lund T, Raymond G, et al Long-term outcomes after allogeneic hematopoietic stem cell transplantation for metachromatic leukodystrophy: the largest single-institution cohort report. Orphanet J Rare Dis. 2015;10:94 Epub 2015/08/08. doi: 10.1186/s13023-015-0313-y [pii]. ; PubMed Central PMCID: PMC4545855. 26245762 10.1186/s13023-015-0313-y PMC4545855

[6] Biffi A, Capotondo A, Fasano S, del Carro U, Marchesini S, Azuma H, et al Gene therapy of metachromatic leukodystrophy reverses neurological damage and deficits in mice. J Clin Invest. 2006;116(11):3070–82. Epub 2006/11/03. doi: 10.1172/JCI28873 ; PubMed Central PMCID: PMC1626132. 17080200 10.1172/JCI28873 PMC1626132

[7] Piguet F, Sondhi D, Piraud M, Fouquet F, Hackett NR, Ahouansou O, et al Correction of brain oligodendrocytes by AAVrh.10 intracerebral gene therapy in metachromatic leukodystrophy mice. Hum Gene Ther. 2012;23(8):903–14. Epub 2012/05/31. doi: 10.1089/hum.2012.015 ; PubMed Central PMCID: PMC3413898. 22642214 10.1089/hum.2012.015 PMC3413898

[8] Biffi A, Montini E, Lorioli L, Cesani M, Fumagalli F, Plati T, et al Lentiviral hematopoietic stem cell gene therapy benefits metachromatic leukodystrophy. Science. 2013;341(6148):1233158 Epub 2013/07/13. doi: 10.1126/science.1233158 1233158 [pii]. . 23845948 10.1126/science.1233158

[9] Sessa M, Lorioli L, Fumagalli F, Acquati S, Redaelli D, Baldoli C, et al Lentiviral haemopoietic stem-cell gene therapy in early-onset metachromatic leukodystrophy: an ad-hoc analysis of a non-randomised, open-label, phase 1/2 trial. The Lancet. 2016;388(10043):476–87. doi: 10.1016/S0140-6736(16)30374-9 10.1016/S0140-6736(16)30374-9 27289174

[10] Beck M. Therapy for lysosomal storage disorders. IUBMB Life. 2010;62(1):33–40. doi: 10.1002/iub.284 . 20014233 10.1002/iub.284

[11] Mechler K, Mountford WK, Hoffmann GF, Ries M. Pressure for drug development in lysosomal storage disorders—a quantitative analysis thirty years beyond the US orphan drug act. Orphanet J Rare Dis. 2015;10:46 Epub 2015/04/22. doi: 10.1186/s13023-015-0262-5 ; PubMed Central PMCID: PMC4404669. 25896727 10.1186/s13023-015-0262-5 PMC4404669

[12] Calias P, Banks WA, Begley D, Scarpa M, Dickson P. Intrathecal delivery of protein therapeutics to the brain: a critical reassessment. Pharmacol Ther. 2014;144(2):114–22. Epub 2014/05/24. doi: S0163-7258(14)00104-1 [pii] doi: 10.1016/j.pharmthera.2014.05.009 . 24854599 10.1016/j.pharmthera.2014.05.009

[13] Pfeifer RW, Felice BR, Boyd RB, Butt MT, Ruiz JA, Heartlein MW, et al Safety evaluation of chronic intrathecal administration of heparan N-sulfatase in juvenile cynomolgus monkeys. Drug Deliv Transl Res. 2012;2(3):187–200. Epub 2012/06/01. doi: 10.1007/s13346-011-0043-1 . 25786866 10.1007/s13346-011-0043-1

[14] Wittke D, Hartmann D, Gieselmann V, Lullmann-Rauch R. Lysosomal sulfatide storage in the brain of arylsulfatase A-deficient mice: cellular alterations and topographic distribution. Acta Neuropathol. 2004;108(4):261–71. Epub 2004/08/24. doi: 10.1007/s00401-004-0883-6 . 15322834 10.1007/s00401-004-0883-6

[15] Calias P, Papisov M, Pan J, Savioli N, Belov V, Huang Y, et al CNS penetration of intrathecal-lumbar idursulfase in the monkey, dog and mouse: implications for neurological outcomes of lysosomal storage disorder. PLoS One. 2012;7(1):e30341 Epub 2012/01/27. doi: 10.1371/journal.pone.0030341 PONE-D-11-15829 [pii]. ; PubMed Central PMCID: PMC3261205. 22279584 10.1371/journal.pone.0030341 PMC3261205

[16] ClinicalTrials.gov. Multicenter Study of HGT-1110 Administered Intrathecally in Children With Metachromatic Leukodystrophy (MLD) (IDEAMLD). Available: https://clinicaltrials.gov/ct2/show/NCT01510028.

[17] Dali C, Sevin C, Riethmueller J, Giugliani R, Troedson C, Bhargava P, et al Intrathecal delivery of recombinant human arylsulfatase A in children with late-infantile metachromatic leukodystrophy. Mol Gen Metab. 2016;117:S14–S124. 10.1016/j.ymgme.2020.07.002 32792226

[18] ClinicalTrials.gov. Open-Label Extension Study Evaluating Safety and Efficacy of HGT-1110 in Patients With Metachromatic Leukodystrophy. Available: https://clinicaltrials.gov/ct2/show/NCT01887938.

[19] U.S., Food Drug Administration. Good laboratory practice for nonclinical laboratory studies 2015. Available: http://www.accessdata.fda.gov/scripts/cdrh/cfdocs/cfcfr/CFRsearch.cfm?CFRPart=58.

[20] Park EI, Mi Y, Unverzagt C, Gabius HJ, Baenziger JU. The asialoglycoprotein receptor clears glycoconjugates terminating with sialic acid alpha 2,6GalNAc. Proc Natl Acad Sci U S A. 2005;102(47):17125–9. doi: 10.1073/pnas.0508537102 ; PubMed Central PMCID: PMCPMC1288006. 16286643 10.1073/pnas.0508537102 PMC1288006

[21] Steirer LM, Park EI, Townsend RR, Baenziger JU. The asialoglycoprotein receptor regulates levels of plasma glycoproteins terminating with sialic acid alpha2,6-galactose. J Biol Chem. 2009;284(6):3777–83. doi: 10.1074/jbc.M808689200 ; PubMed Central PMCID: PMCPMC2635048. 19075021 10.1074/jbc.M808689200 PMC2635048

[22] Matzner U, Lullmann-Rauch R, Stroobants S, Andersson C, Weigelt C, Eistrup C, et al Enzyme replacement improves ataxic gait and central nervous system histopathology in a mouse model of metachromatic leukodystrophy. Mol Ther. 2009;17(4):600–6. Epub 2009/01/29. doi: mt2008305 [pii] doi: 10.1038/mt.2008.305 ; PubMed Central PMCID: PMC2835113. 19174759 10.1038/mt.2008.305 PMC2835113

[23] Ghosh P, Dahms NM, Kornfeld S. Mannose 6-phosphate receptors: new twists in the tale. Nat Rev Mol Cell Biol. 2003;4(3):202–12. doi: 10.1038/nrm1050 . 12612639 10.1038/nrm1050

[24] International Conference on Harmonisation. International Conference on Harmonisation; guidance on Q5E comparability of biotechnological/biological products subject to changes in their manufacturing process. Fed Regist. 2005;70(125):37861–2. Epub 2005/07/02. . 15988855

[25] U.S. FDA. Guidance for Industry: CGMP for Phase 1 Investigational Drugs (2008). Available from: http://www.fda.gov/downloads/Drugs/GuidanceComplianceRegulatoryInformation/Guidances/ucm070273.pdf.

[26] Matzner U, Matthes F, Herbst E, Lullmann-Rauch R, Callaerts-Vegh Z, D'Hooge R, et al Induction of tolerance to human arylsulfatase A in a mouse model of metachromatic leukodystrophy. Mol Med. 2007;13(9–10):471–9. Epub 2007/07/31. doi: 10.2119/2007-00063.Matzner ; PubMed Central PMCID: PMC1933260. 17660863 10.2119/2007-00063.Matzner PMC1933260

[27] Meikle PJ, Brooks DA, Ravenscroft EM, Yan M, Williams RE, Jaunzems AE, et al Diagnosis of lysosomal storage disorders: evaluation of lysosome-associated membrane protein LAMP-1 as a diagnostic marker. Clin Chem. 1997;43(8 Pt 1):1325–35. Epub 1997/08/01. . 9267309

[28] The use of non-human primates in research. A working group report chaired by Sir David Weatherall. Available from: http://www.acmedsci.ac.uk/viewFile/publicationDownloads/1165861003.pdf.

[29] Shire. A phase I/II multicenter open-label dose escalation study of HGT-1110 administered intrathecally in children with metachromatic leukodystrophy (NCT01510028) 2011 [cited 2016 21 March]. Available from: https://clinicaltrials.gov/ct2/show/NCT01510028.

[30] Shire. An open-label extension of study HGT-MLD-070 evaluating long term safety and efficacy of intrathecal administration of HGT-1110 in patients with metachromatic leukodystrophy (NCT01887938) 2013 [cited 2016 21 March]. Available from: https://clinicaltrials.gov/ct2/show/NCT01887938.

[31] Dekaban AS. Changes in brain weights during the span of human life: relation of brain weights to body heights and body weights. Ann Neurol. 1978;4(4):345–56. Epub 1978/10/01. doi: 10.1002/ana.410040410 . 727739 10.1002/ana.410040410

[32] Felice BR, Wright TL, Boyd RB, Butt MT, Pfeifer RW, Pan J, et al Safety evaluation of chronic intrathecal administration of idursulfase-IT in cynomolgus monkeys. Toxicol Pathol. 2011;39(5):879–92. doi: 10.1177/0192623311409595 . 21628718 10.1177/0192623311409595

[33] Neufeld EF. Lysosomal Storage Diseases. Annual Review of Biochemistry. 1991;60(1):257–80. doi: 10.1146/annurev.bi.60.070191.001353 . 1883197 10.1146/annurev.bi.60.070191.001353

[34] Gabrielsson J, Weiner D. Pharmacokinetic and Pharmacodynamic Data Analysis, Concepts and Applications. 4th Edition Sweden: Swedish Pharmaceutical Press; 2007.

[35] Salamat-Miller N, Taylor K, Campolieto P, Shahrokh Z, Pan J, Charnas L, et al. Methods and compositions for CNS delivery of arylsulfatase A. Google Patents; 2013. Available: https://www.google.com/patents/EP2585104A2?cl=en.

