# Supplementary figures and images for: Nonclinical comparability studies of recombinant human arylsulfatase A addressing manufacturing process changes
Source: PLoS One. 2018 Apr 19;13(4):e0195186. doi: 10.1371/journal.pone.0195186 (PMC5908175; doi:10.1371/journal.pone.0195186)

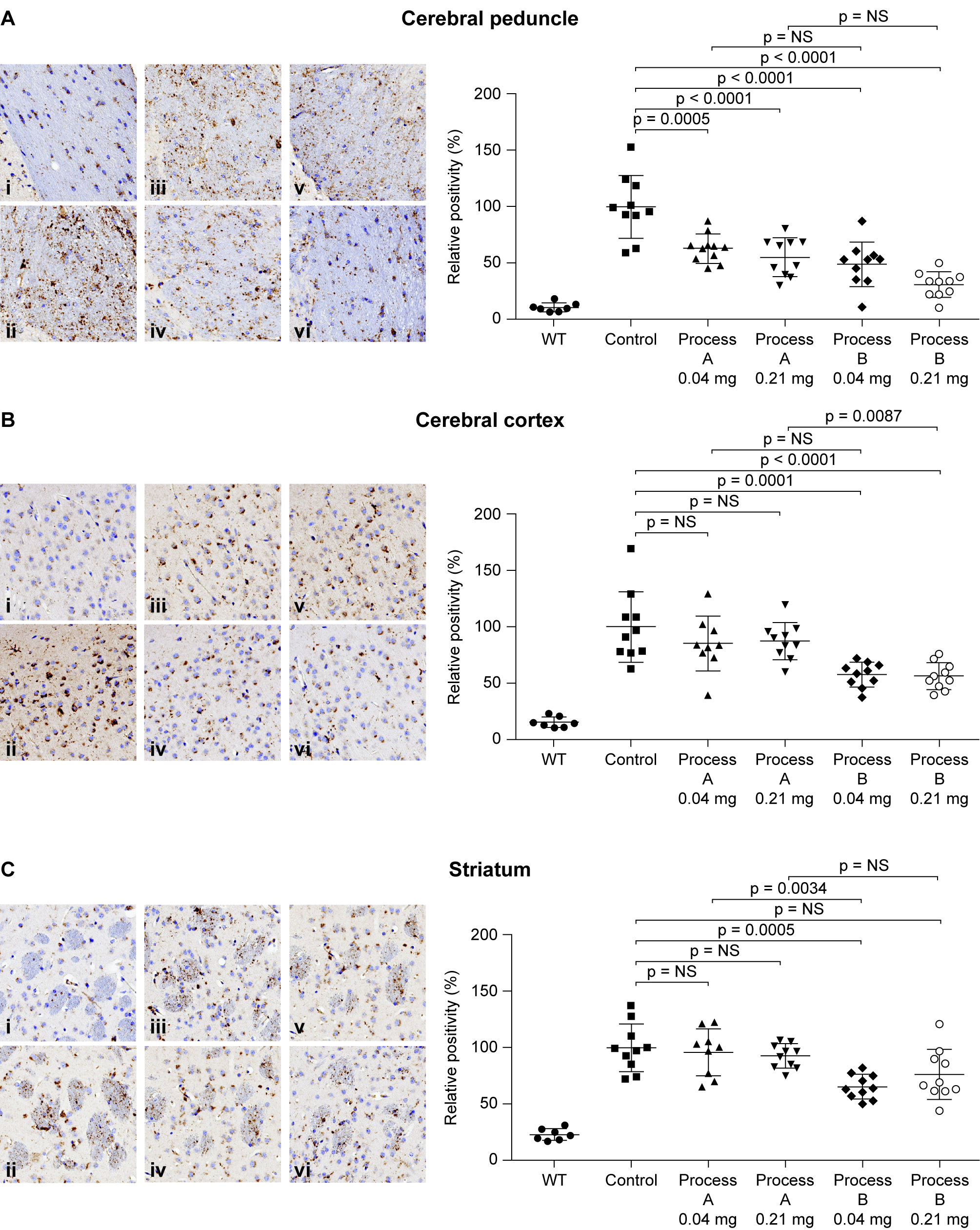

Supplement: S1 Fig — Representative images of immunohistochemical staining of LAMP-1 with corresponding morphometric analysis in the (A) cerebral peduncle, (B) cerebral cortex and (C) striatum of immunotolerant MLD mice treated with rhASA 0.04 mg or 0.21 mg from process A (iii, v) or process B (iv, vi), or control (ii). Untreated C57/B16 mice served as WT controls (i). Individual and mean values can be found in S2 Table. LAMP-1, lysosomal-associated membrane protein-1; MLD, metachromatic leukodystrophy; NS, not significant; rhASA, recombinant human arylsulfatase A; WT, wild type. (TIF) [file pone.0195186.s001.tif]
