# Supplementary material for: Nonclinical comparability studies of recombinant human arylsulfatase A addressing manufacturing process changes
Source: PLoS One. 2018 Apr 19;13(4):e0195186. doi: 10.1371/journal.pone.0195186 (PMC5908175; doi:10.1371/journal.pone.0195186)
Supplement: S1 Table — LAMP-1, lysosomal-associated membrane protein-1; MLD, metachromatic leukodystrophy; rhASA, recombinant human arylsulfatase A; SD, standard deviation; WT, wild-type. (DOCX) [file pone.0195186.s002.docx]

**S1 Table.** **Morphometry analysis of LAMP-1 staining in white matter of spinal cord and regions of the brain** **of immunotolerant MLD mice treated with rhASA 0.04 mg or 0.21 mg from process A or process B or control.**

|  |  | LAMP-1 positivity (%) | | | | | | |
| --- | --- | --- | --- | --- | --- | --- | --- | --- |
| **Region** | **Animal** | **WT*** | Control | | Process A | | Process B | |
|  |  |  | Untreated | Vehicle | 0.04 mg | 0.21 mg | 0.04 mg | 0.21 mg |
| Spinal cord | 1 | 0.50 | 2.11 | – | 1.81 | 1.38 | 1.15 | 1.01 |
|  | 2 | 0.48 | 2.47 | – | 1.61 | 1.03 | 1.59 | 2.02 |
|  | 3 | 0.44 | 3.14 | – | 1.63 | 1.58 | 2.12 | 1.59 |
|  | 4 | 0.34 | 2.44 | – | – | 1.47 | 1.49 | 1.07 |
|  | 5 | 0.43 | 2.89 | – | 1.91 | 1.02 | 1.64 | 1.03 |
|  | 6 | 0.43 | 2.27 | – | 1.47 | 0.77 | 1.65 | 0.85 |
|  | 7 | 0.29 | – | 2.70 | 1.48 | 1.67 | 1.96 | 1.18 |
|  | 8 | – | – | 3.27 | 2.86 | 1.72 | 2.64 | 1.69 |
|  | 9 | – | – | 2.37 | 2.66 | 2.16 | 3.40 | 1.05 |
|  | 10 | – | – | 3.98 | 2.44 | 2.44 | 2.47 | 1.45 |
|  | Mean | 0.41 | 2.76 | | 1.98 | 1.52 | 2.01 | 1.29 |
|  | SD | 0.07 | 0.57 | | 0.53 | 0.52 | 0.67 | 0.38 |
| Cerebella | 1 | 0.77 | 8.13 | – | 2.84 | 3.55 | 4.03 | 4.14 |
|  | 2 | 0.73 | 7.21 | – | 3.77 | 4.06 | 2.66 | 3.51 |
|  | 3 | 0.76 | 4.80 | – | 5.22 | 2.65 | 3.23 | 2.66 |
|  | 4 | 0.49 | 5.43 | – | – | 4.93 | 3.65 | 2.44 |
|  | 5 | 0.39 | 5.68 | – | 3.65 | 5.82 | 3.48 | 2.53 |
|  | 6 | 0.85 | 4.55 | – | 2.92 | 3.79 | 4.71 | 3.94 |
|  | 7 | 0.85 | – | 4.10 | 4.44 | 3.18 | 4.37 | 2.93 |
|  | 8 | – | – | 4.20 | 5.07 | 3.80 | 4.06 | 3.45 |
|  | 9 | – | – | 5.88 | 3.39 | 4.14 | 5.68 | 2.01 |
|  | 10 | – | – | 5.34 | 3.99 | 3.64 | 4.17 | 4.15 |
|  | Mean | 0.69 | 5.53 | | 3.92 | 3.96 | 4.00 | 3.18 |
|  | SD | 0.18 | 1.29 | | 0.85 | 0.89 | 0.84 | 0.77 |
| Fimbria | 1 | 0.24 | 5.46 | – | 2.55 | 2.30 | 2.86 | 2.08 |
|  | 2 | 0.47 | 4.71 | – | 2.08 | 3.91 | 1.70 | 3.82 |
|  | 3 | 0.40 | 2.90 | – | 2.74 | 3.26 | 2.14 | 1.82 |
|  | 4 | 0.26 | 4.33 | – | – | 3.07 | 1.60 | 1.94 |
|  | 5 | 0.21 | 4.22 | – | 2.51 | 2.59 | 2.10 | 1.49 |
|  | 6 | 0.54 | 3.25 | – | 1.31 | 5.04 | 3.41 | 4.43 |
|  | 7 | 0.55 | – | 3.25 | 2.82 | 4.20 | 2.88 | 2.06 |
|  | 8 | – | – | 4.03 | 2.66 | 3.47 | 2.99 | 2.99 |
|  | 9 | – | – | 3.16 | 2.60 | 3.85 | 4.19 | 3.25 |
|  | 10 | – | – | 3.14 | 2.77 | 2.88 | 2.34 | 3.10 |
|  | Mean | 0.38 | 3.85 | | 2.45 | 3.46 | 2.62 | 2.70 |
|  | SD | 0.14 | 0.84 | | 0.48 | 0.82 | 0.80 | 0.97 |

* Untreated C57/B16 mice served as WT controls.

LAMP-1, lysosomal-associated membrane protein-1; MLD, metachromatic leukodystrophy; rhASA, recombinant human arylsulfatase A; SD, standard deviation; WT, wild-type.
