# Supplementary material for: Nonclinical comparability studies of recombinant human arylsulfatase A addressing manufacturing process changes
Source: PLoS One. 2018 Apr 19;13(4):e0195186. doi: 10.1371/journal.pone.0195186 (PMC5908175; doi:10.1371/journal.pone.0195186)
Supplement: S2 Table — LAMP-1, lysosomal-associated membrane protein-1; MLD, metachromatic leukodystrophy; rhASA, recombinant human arylsulfatase A; SD, standard deviation; WT, wild-type. (DOCX) [file pone.0195186.s003.docx]

**S2 Table.** **Morphometry analysis of LAMP-1 staining in additional regions of the brain** **of immunotolerant MLD mice treated with rhASA 0.04 mg or 0.21 mg from process A or process B or control.**

|  |  | LAMP-1 positivity (%) | | | | | | |
| --- | --- | --- | --- | --- | --- | --- | --- | --- |
| **Region** | **Animal** | **WT*** | Control | | Process A | | Process B | |
|  |  |  | Untreated | Vehicle | 0.04 mg | 0.21 mg | 0.04 mg | 0.21 mg |
| Cerebral peduncle | 1 | 0.50 | 5.99 | – | 3.33 | 1.90 | 2.87 | 1.11 |
|  | 2 | 0.36 | 7.71 | – | 4.41 | 1.53 | 1.82 | 1.71 |
|  | 3 | 0.57 | 5.10 | – | 3.93 | 3.29 | 3.05 | 1.72 |
|  | 4 | 0.49 | 4.81 | – | – | 3.46 | 2.67 | 1.14 |
|  | 5 | 0.69 | 6.23 | – | 3.31 | 3.44 | 1.73 | 1.27 |
|  | 6 | 0.35 | 4.64 | – | 2.31 | 3.44 | 0.57 | 0.55 |
|  | 7 | 0.94 | – | 4.99 | 2.87 | 1.99 | 2.29 | 1.91 |
|  | 8 | – | – | 4.65 | 2.72 | 2.41 | 2.67 | 2.07 |
|  | 9 | – | – | 3.17 | 3.23 | 4.05 | 4.40 | 1.68 |
|  | 10 | – | – | 2.98 | 2.44 | 2.32 | 2.71 | 2.52 |
|  | Mean | 0.56 | 5.03 | | 3.17 | 2.78 | 2.48 | 1.57 |
|  | SD | 0.21 | 1.40 | | 0.68 | 0.85 | 1.00 | 0.56 |
| Cerebral cortex | 1 | 0.29 | 1.54 | – | 1.37 | 1.02 | 0.98 | 1.02 |
|  | 2 | 0.22 | 1.11 | – | 1.10 | 1.19 | 0.73 | 0.76 |
|  | 3 | 0.21 | 1.55 | – | 1.85 | 1.29 | 0.75 | 0.80 |
|  | 4 | 0.15 | 1.30 | – | – | 1.20 | 0.54 | 0.86 |
|  | 5 | 0.18 | 1.84 | – | 1.20 | 1.31 | 0.65 | 0.60 |
|  | 6 | 0.33 | 1.09 | – | 0.57 | 1.37 | 0.94 | 0.75 |
|  | 7 | 0.16 | – | 0.90 | 1.16 | 1.41 | 0.91 | 0.68 |
|  | 8 | – | – | 2.42 | 1.46 | 1.71 | 0.84 | 0.93 |
|  | 9 | – | – | 1.38 | 1.04 | 1.10 | 1.04 | 0.56 |
|  | 10 | – | – | 1.12 | 1.20 | 0.86 | 0.87 | 1.08 |
|  | Mean | 0.22 | 1.43 | | 1.21 | 1.24 | 0.82 | 0.80 |
|  | SD | 0.07 | 0.45 | | 0.35 | 0.23 | 0.16 | 0.17 |
| Striatum | 1 | 0.21 | 1.26 | – | 1.40 | 0.94 | 0.65 | 0.76 |
|  | 2 | 0.32 | 1.13 | – | 1.11 | 1.11 | 0.57 | 0.51 |
|  | 3 | 0.25 | 1.56 | – | 1.38 | 1.10 | 0.69 | 0.98 |
|  | 4 | 0.19 | 0.97 | – | – | 0.86 | 0.73 | 0.79 |
|  | 5 | 0.22 | 1.14 | – | 1.17 | 1.20 | 0.60 | 0.72 |
|  | 6 | 0.36 | 0.82 | – | 0.75 | 1.21 | 0.72 | 1.38 |
|  | 7 | 0.29 | – | 1.06 | 1.14 | 0.94 | 0.80 | 0.70 |
|  | 8 | – | – | 1.46 | 1.20 | 1.16 | 0.94 | 0.70 |
|  | 9 | – | – | 1.11 | 0.79 | 1.05 | 0.88 | 1.11 |
|  | 10 | – | – | 0.84 | 0.88 | 0.97 | 0.86 | 1.03 |
|  | Mean | 0.26 | 1.14 | | 1.09 | 1.05 | 0.74 | 0.87 |
|  | SD | 0.06 | 0.24 | | 0.24 | 0.12 | 0.12 | 0.25 |

* Untreated C57/B16 mice served as WT controls.

LAMP-1, lysosomal-associated membrane protein-1; MLD, metachromatic leukodystrophy; rhASA, recombinant human arylsulfatase A; SD, standard deviation; WT, wild-type.
