# Supplementary material for: Nonclinical comparability studies of recombinant human arylsulfatase A addressing manufacturing process changes
Source: PLoS One. 2018 Apr 19;13(4):e0195186. doi: 10.1371/journal.pone.0195186 (PMC5908175; doi:10.1371/journal.pone.0195186)
Supplement: S3 Table — F, female; h, hour; M, male; rhASA, recombinant human arylsulfatase A. SD, standard deviation. (DOCX) [file pone.0195186.s004.docx]

**S3 Table.** **Individual and mean serum concentrations (ng/mL) of rhASA in juvenile cynomolgus monkeys following intrathecal administration of rhASA 6.0 mg manufactured using process A or process B.**

|  |  |  | **Time after dose (h)** | | | | | | | |
| --- | --- | --- | --- | --- | --- | --- | --- | --- | --- | --- |
| **Process** | **Sex** | **Animal** | **0.083** | **0.25** | **0.5** | **1** | **2** | **4** | **8** | **24** |
| A | M | 1 | 0 | 0 | 0 | 0 | 80.7 | 271 | 325 | 62.3 |
|  |  | 2 | 0 | 45.5 | 83.5 | 127 | 265 | 583 | 334 | 74.3 |
|  |  | 3 | 0 | 0 | 0 | 43.2 | 264 | 392 | 393 | 61.3 |
|  |  | 4 | 45.8 | 0 | 0 | 0 | 90.4 | 515 | 164 | 0 |
|  |  | 5 | 51.5 | 0 | 0 | 48.5 | 275 | 651 | 400 | 0 |
|  | F | 7 | 0 | 88.9 | 126 | 327 | 524 | 393 | 272 | 53.5 |
|  |  | 8 | 0 | 0 | 0 | 67.4 | 523 | 605 | 237 | 0 |
|  |  | 9 | 0 | 0 | 0 | 71.2 | 823 | 531 | 521 | 88.0 |
|  |  | 10 | 67.8 | 84.7 | 65.4 | 62.0 | 208 | 606 | 360 | 69.3 |
|  |  | Mean | 55.0 | 73.0 | 91.6 | 107 | 339 | 505 | 334 | 68.1 |
| B | M | 1 | 0 | 0 | 0 | 0 | 153 | 549 | 456 | 76.6 |
|  |  | 2 | 0 | 0 | 49.5 | 151 | 1070 | 977 | 448 | 0 |
|  |  | 3 | 0 | 0 | 0 | 0 | 268 | 660 | 432 | 0 |
|  |  | 4 | 0 | 0 | 0 | 44.1 | 323 | 560 | 313 | 47.4 |
|  |  | 5 | 0 | 0 | 0 | 45.3 | 399 | 703 | 393 | 56.6 |
|  |  | 6 | 0 | 0 | 0 | 87.9 | 279 | 594 | 354 | 0 |
|  | F | 7 | 0 | 0 | 0 | 0 | 71.9 | 325 | 244 | 0 |
|  |  | 8 | 0 | 44.8 | 82.4 | 135 | 539 | 616 | 352 | 0 |
|  |  | 9 | 81.1 | 41.2 | 64.5 | 81.5 | 522 | 775 | 648 | 65.3 |
|  |  | 10 | 0 | 59.9 | 64.0 | 62.3 | 182 | 622 | 518 | 71.1 |
|  |  | Mean | 81.1 | 48.6 |  | 86.7 | 381 | 638 | 416 | 63.4 |

F, female; h, hour; M, male; rhASA, recombinant human arylsulfatase A. SD, standard deviation.
