# Supplementary material for: Nonclinical comparability studies of recombinant human arylsulfatase A addressing manufacturing process changes
Source: PLoS One. 2018 Apr 19;13(4):e0195186. doi: 10.1371/journal.pone.0195186 (PMC5908175; doi:10.1371/journal.pone.0195186)
Supplement: S4 Table — CSF, cerebrospinal fluid; F, female; h, hour; M, male; rhASA, recombinant human arylsulfatase A; SD, standard deviation. (DOCX) [file pone.0195186.s005.docx]

**S4 Table.** **Individual and mean CSF concentrations (ng/mL) of rhASA in juvenile cynomolgus monkeys following intrathecal administration of rhASA 6.0 mg manufactured using process A or process B.**

|  |  |  | **Time after dose (h)** | | | | | | | | | | |
| --- | --- | --- | --- | --- | --- | --- | --- | --- | --- | --- | --- | --- | --- |
| **Process** | **Sex** | **Animal** | **0** | **0.083** | **0.25** | **0.5** | **1** | **2** | **4** | **8** | **24** | **48** | **72** |
| A | M | 1 | 0 | 636000 | 551000 | 393000 | 269000 | 99300 | 89800 | 31900 | 2140 | 661 | 970 |
|  |  | 2 | 0 | 99500 | 555000 | 411000 | 243000 | 123000 | 75400 | 19900 | 950 | 1090 | 1130 |
|  |  | 3 | 0 | 925000 | 718000 | 434000 | 335000 | 198000 | 162000 | 53200 | 4350 | 416 | 129 |
|  |  | 4 | 40.5 | 454000 | 430000 | 342000 | 228000 | 134000 | 49500 | 10400 | 2750 | 298 | 162 |
|  |  | 5 | 101 | 682000 | 588000 | 179000 | 292000 | 188000 | 10400 | 44700 | 4740 | 639 | 82.1 |
|  | F | 7 | 0 | 510000 | 374000 | 206000 | 187000 | 115000 | 84000 | 31400 | 1160 | 784 | 346 |
|  |  | 8 | 214 | 990000 | 664000 | 518000 | 318000 | 207000 | 72300 | 22700 | 2180 | 0 | 0 |
|  |  | 9 | 103 | 644000 | 615000 | 472000 | 266000 | 162000 | 90200 | 37300 | 7560 | 1610 | 447 |
|  |  | 10 | 0 | 482000 | 459000 | 333000 | 203000 | 140000 | 79200 | 31500 | 2960 | 756 | 559 |
|  |  | Mean | 115 | 702000 | 550000 | 365000 | 260000 | 152000 | 89600 | 31400 | 3200 | 782 | 478 |
| B | M | 1 | 149 | 993000 | 688000 | 555000 | 376000 | 214000 | 116000 | 46900 | 6510 | 578 | 370 |
|  |  | 2 | 177 | 526000 | 280000 | 297000 | 187000 | 124000 | 56900 | 51200 | 2020 | 2390 | 626 |
|  |  | 3 | 115 | 492000 | 350000 | 322000 | 152000 | 171000 | 97400 | 37100 | 3360 | 941 | 894 |
|  |  | 4 | 0 | 519000 | 370000 | 193000 | 170000 | 95100 | 81800 | 25500 | 1220 | 2130 | 337 |
|  |  | 5 | 0 | 167000 | 418000 | 259000 | 345000 | 202000 | 143000 | 74300 | 4900 | 534 | 273 |
|  |  | 6 | 0 | 938000 | 637000 | 475000 | 307000 | 156000 | 131000 | 13900 | 0 | 0 | 0 |
|  | F | 7 | 41.4 | 765000 | 488000 | 404000 | 227000 | 141000 | 78600 | 35400 | 5150 | 1000 | 297 |
|  |  | 8 | 0 | 756000 | 315000 | 217000 | 161000 | 76800 | 43400 | 18500 | 2310 | 343 | 193 |
|  |  | 9 | 0 | 624000 | 302000 | 254000 | 166000 | 92500 | 88700 | 61100 | 8760 | 1720 | 649 |
|  |  | 10 | 336 | 195000 | 306000 | 201000 | 182000 | 128000 | 40700 | 25700 | 10800 | 736 | 1490 |
|  |  | Mean | 164 | 598000 | 415000 | 318000 | 227000 | 140000 | 87800 | 39000 | 5000 | 1150 | 570 |

CSF, cerebrospinal fluid; F, female; h, hour; M, male; rhASA, recombinant human arylsulfatase A; SD, standard deviation.
