# Supplementary material for: Nonclinical comparability studies of recombinant human arylsulfatase A addressing manufacturing process changes
Source: PLoS One. 2018 Apr 19;13(4):e0195186. doi: 10.1371/journal.pone.0195186 (PMC5908175; doi:10.1371/journal.pone.0195186)
Supplement: S5 Table — AUCinf, area under the concentration–time curve from time 0 to infinity; AUClast, area under the concentration–time curve from time 0 to the last measurement; CL, clearance; Cmax, maximum plasma concentration; CSF, cerebrospinal fluid; F, female; λz, terminal rate constant; h, hour; M, male; MRTinf, mean residence time to infinity; n, number; NC, not calculated; t½, terminal elimination phase half-life; rhASA, recombinant human arylsulfatase A; SD, standard deviation; Tmax, time to maximum plasma concentration; Vz, volume of distribution. (DOCX) [file pone.0195186.s006.docx]

**S5 Table.** **Individual and mean CSF pharmacokinetic parameters in juvenile cynomolgus monkeys following intrathecal lumbar administration of rhASA 6.0 mg manufactured using process A or process B.**

| **Process** | **Sex** | **Animal** | λz, L/h | t_½_, h | T_max_, h | C_max_, ng/mL | AUC_last_, h·ng/mL | AUC_inf_, h·ng/mL | Vz, mL | CL, mL/h | MRT_inf_, h |
| --- | --- | --- | --- | --- | --- | --- | --- | --- | --- | --- | --- |
| A | M | 1 | NC | NC | 0.0833 | 636000 | 1380000 | NC | NC | NC | NC |
|  |  | 2 | NC | NC | 0.0833 | 995000 | 1300000 | NC | NC | NC | NC |
|  |  | 3 | 0.113 | 6.16 | 0.0833 | 925000 | 2140000 | 2140000 | 24.9 | 2.81 | 4.65 |
|  |  | 4 | 0.0720 | 9.63 | 0.0833 | 454000 | 980000 | 981000 | 84.9 | 6.11 | 4.01 |
|  |  | 5 | 0.117 | 5.90 | 0.0833 | 682000 | 1660000 | 1670000 | 30.6 | 3.60 | 5.08 |
|  | F | 7 | 0.0252 | 27.5 | 0.0833 | 510000 | 1170000 | 1180000 | 201 | 5.07 | 6.23 |
|  |  | 8 | 0.172 | 4.03 | 0.0833 | 990000 | 1510000 | 1520000 | 22.9 | 3.94 | 2.88 |
|  |  | 9 | 0.0641 | 10.8 | 0.0833 | 644000 | 1690000 | 1690000 | 55.3 | 3.55 | 6.47 |
|  |  | 10 | 0.0848 | 8.18 | 0.0833 | 482000 | 1300000 | 1310000 | 54.2 | 4.59 | 5.71 |
|  |  | Mean | 0.0926 | 10.3 | 0.0833 | 702000 | 1460000 | 1500000 | 67.7 | 4.24 | 5.00 |
|  |  | SD | 0.0469 | 7.93 | 0.00 | 216000 | 340000 | 383000 | 62.9 | 1.11 | 1.28 |
| B | M | 1 | 0.100 | 6.93 | 0.0833 | 993000 | 2080000 | 2090000 | 28.8 | 2.88 | 4.85 |
|  |  | 2 | 0.0884 | 7.84 | 0.0833 | 526000 | 1370000 | 1370000 | 49.5 | 4.37 | 6.99 |
|  |  | 3 | 0.0898 | 7.72 | 0.0833 | 492000 | 1410000 | 1410000 | 47.2 | 4.24 | 6.18 |
|  |  | 4 | NC | NC | 0.0833 | 519000 | 1090000 | NC | NC | NC | NC |
|  |  | 5 | 0.108 | 6.43 | 0.250 | 418000 | 2060000 | 2060000 | 27.0 | 2.91 | 5.82 |
|  |  | 6 | 0.456 | 1.52 | 0.0833 | 938000 | 1360000 | 1390000 | 9.46 | 4.31 | 2.11 |
|  | F | 7 | 0.0624 | 11.1 | 0.0833 | 765000 | 1490000 | 1490000 | 64.5 | 4.02 | 5.77 |
|  |  | 8 | 0.0862 | 8.04 | 0.0833 | 756000 | 898000 | 900000 | 77.3 | 6.66 | 4.75 |
|  |  | 9 | 0.0542 | 12.8 | 0.0833 | 624000 | 1640000 | 1650000 | 67.1 | 3.64 | 8.64 |
|  |  | 10 | NC | NC | 0.250 | 306000 | 1110000 | NC | NC | NC | NC |
|  |  | Mean | 0.131 | 7.80 | 0.117 | 634000 | 1450000 | 1550000 | 46.3 | 4.13 | 5.64 |
|  |  | SD | 0.133 | 3.34 | 0.0703 | 224000 | 391000 | 389000 | 23.2 | 1.19 | 1.89 |

AUC_inf_, area under the concentration–time curve from time 0 to infinity; AUC_last_, area under the concentration–time curve from time 0 to the last measurement; CL, clearance; C_max_, maximum plasma concentration; CSF, cerebrospinal fluid; F, female; λz, terminal rate constant; h, hour; M, male; MRT_inf_, mean residence time to infinity; n, number; NC, not calculated; t_½_, terminal elimination phase half-life; rhASA, recombinant human arylsulfatase A; SD, standard deviation; T_max_, time to maximum plasma concentration; Vz, volume of distribution.
