# Supplementary material for: Nonclinical comparability studies of recombinant human arylsulfatase A addressing manufacturing process changes
Source: PLoS One. 2018 Apr 19;13(4):e0195186. doi: 10.1371/journal.pone.0195186 (PMC5908175; doi:10.1371/journal.pone.0195186)
Supplement: S6 Table — λz, t½, Vz, CL and MRTinf could not be determined in serum samples. AUClast, area under the concentration–time curve from time 0 to the last measurement; h, hour; rhASA, recombinant human arylsulfatase A; SD, standard deviation; Tmax, time to maximum plasma concentration. (DOCX) [file pone.0195186.s007.docx]

**S6 Table.** **Individual and mean serum pharmacokinetic parameters in juvenile cynomolgus monkeys following intrathecal lumbar administration of rhASA 6.0 mg manufactured using process A or process B.**

| **Process** | **Sex** | **Animal** | T_max_, h | C_max_, ng/mL | AUC_last_, h·ng/mL |
| --- | --- | --- | --- | --- | --- |
| A | M | 1 | 8.00 | 325 | 4720 |
|  |  | 2 | 4.00 | 583 | 6220 |
|  |  | 3 | 8.00 | 393 | 6040 |
|  |  | 4 | 4.00 | 515 | 2050 |
|  |  | 5 | 4.00 | 651 | 3210 |
|  | F | 7 | 2.00 | 524 | 5430 |
|  |  | 8 | 4.00 | 605 | 3140 |
|  |  | 9 | 2.00 | 823 | 8810 |
|  |  | 10 | 4.00 | 606 | 6380 |
|  |  | Mean | 4.44 | 558 | 5110 |
|  |  | SD | 2.19 | 145 | 2080 |
| B | M | 1 | 4.00 | 549 | 7130 |
|  |  | 2 | 2.00 | 1070 | 5570 |
|  |  | 3 | 4.00 | 660 | 3380 |
|  |  | 4 | 4.00 | 560 | 5720 |
|  |  | 5 | 4.00 | 703 | 7140 |
|  |  | 6 | 4.00 | 594 | 3000 |
|  | F | 7 | 4.00 | 325 | 1610 |
|  |  | 8 | 4.00 | 616 | 3500 |
|  |  | 9 | 4.00 | 775 | 10200 |
|  |  | 10 | 4.00 | 622 | 7970 |
|  |  | Mean | 3.80 | 647 | 5520 |
|  |  | SD | 0.632 | 190 | 2660 |

λz, t_½_, Vz, CL and MRT_inf_ could not be determined in serum samples.

AUC_last_, area under the concentration–time curve from time 0 to the last measurement; h, hour; rhASA, recombinant human arylsulfatase A; SD, standard deviation; T_max_, time to maximum plasma concentration.
