# Supplementary material for: Nonclinical comparability studies of recombinant human arylsulfatase A addressing manufacturing process changes
Source: PLoS One. 2018 Apr 19;13(4):e0195186. doi: 10.1371/journal.pone.0195186 (PMC5908175; doi:10.1371/journal.pone.0195186)
Supplement: S7 Table — The total excreted in urine was calculated by adding the value for urine, cage rinse and cage wash. rhASA, recombinant human arylsulfatase A; h, hour. (DOCX) [file pone.0195186.s008.docx]

**S7 Table. Recovery of process A and process B [125I]-rhASA 0.62 mg after a single intrathecal dose in male Sprague Dawley rats.**

|  |  | **% of dose recovered** | | | | | | |
| --- | --- | --- | --- | --- | --- | --- | --- | --- |
|  |  | **Process A** | | | **Process B** | | | |
|  | **Collection time, h** | **Animal 1** | **Animal 2** | **Mean** | **Animal 1** | **Animal 2** | **Animal 3** | **Mean** |
| Urine | 0–6 | 15.8 | 21.9 | 18.8 | 19.1 | 19.4 | 21.1 | 19.9 |
|  | 6–12 | 13.2 | 18.7 | 15.9 | 17.6 | 2.72 | 15.4 | 11.9 |
|  | 12–24 | 8.13 | 8.15 | 8.14 | 7.13 | 7.63 | 7.36 | 7.37 |
|  | 24–48 | 10.4 | 9.61 | 10.0 | 9.69 | 8.81 | 9.39 | 9.30 |
|  | 48–72 | 5.20 | 5.73 | 5.46 | 5.93 | 6.73 | 4.67 | 5.78 |
|  | 72–96 | 3.67 | 3.24 | 3.45 | 3.44 | 4.11 | 3.51 | 3.69 |
|  | 96–120 | 2.36 | 2.52 | 2.44 | 2.66 | 3.03 | 2.61 | 2.76 |
|  | 120–144 | 1.54 | 1.56 | 1.55 | 1.11 | 1.89 | 1.76 | 1.59 |
|  | 144–168 | 1.15 | 1.17 | 1.16 | 1.18 | 1.30 | 1.06 | 1.18 |
|  | Subtotal | 61.3 | 72.5 | 66.9 | 67.9 | 55.6 | 66.8 | 63.5 |
| Feces | 0–12 | 0.15 | 0.29 | 0.22 | 0.22 | 0.36 | 0.35 | 0.31 |
|  | 12–24 | 0.65 | 0.92 | 0.79 | 0.48 | 0.46 | 0.54 | 0.49 |
|  | 24–48 | 2.05 | 1.64 | 1.84 | 1.41 | 1.43 | 1.70 | 1.51 |
|  | 48–72 | 1.43 | 1.20 | 1.32 | 1.19 | 1.61 | 0.93 | 1.24 |
|  | 72–96 | 1.09 | 0.90 | 0.99 | 0.87 | 0.84 | 0.66 | 0.79 |
|  | 96–120 | 0.60 | 0.69 | 0.64 | 0.61 | 0.73 | 0.60 | 0.65 |
|  | 120–144 | 0.43 | 0.36 | 0.39 | 0.50 | 0.39 | 0.40 | 0.43 |
|  | 144–168 | 0.27 | 0.20 | 0.24 | 0.26 | 0.31 | 0.32 | 0.30 |
|  | Subtotal | 6.67 | 6.20 | 6.43 | 5.54 | 6.13 | 5.50 | 5.72 |
| Cage rinse | 0–6 | 4.64 | 3.53 | 4.08 | 2.42 | 2.99 | 2.51 | 2.64 |
|  | 6–12 | 1.37 | 1.49 | 1.43 | 0.83 | 0.90 | 1.03 | 0.92 |
|  | 12–24 | 0.77 | 0.76 | 0.76 | 0.94 | 1.34 | 1.08 | 1.12 |
|  | 24–48 | 0.79 | 0.58 | 0.68 | 0.58 | 0.78 | 1.11 | 0.82 |
|  | 48–72 | 0.50 | 0.56 | 0.53 | 0.44 | 0.66 | 0.59 | 0.56 |
|  | 72–96 | 0.44 | 0.33 | 0.38 | 0.33 | 0.50 | 0.48 | 0.44 |
|  | 96–120 | 0.55 | 0.30 | 0.42 | 0.29 | 0.32 | 0.68 | 0.43 |
|  | 120–144 | 0.36 | 0.18 | 0.27 | 0.24 | 0.34 | 0.39 | 0.32 |
|  | 144–168 | 0.20 | 0.13 | 0.17 | 0.17 | 0.21 | 0.31 | 0.23 |
|  | Subtotal | 9.60 | 7.86 | 8.73 | 6.24 | 8.03 | 8.19 | 7.49 |
| Cage wash |  | 0.04 | 0.02 | 0.03 | 0.11 | 0.08 | 1.07 | 0.42 |
| Carcass |  | 8.25 | 7.10 | 7.68 | 5.24 | 7.18 | 5.80 | 6.07 |
| Total |  | 85.9 | 93.7 | 89.8 | 85.1 | 77.0 | 87.4 | 83.2 |

The total excreted in urine was calculated by adding the value for urine, cage rinse and cage wash.

rhASA, recombinant human arylsulfatase A; h, hour
