# Supplementary material for: Nonclinical comparability studies of recombinant human arylsulfatase A addressing manufacturing process changes
Source: PLoS One. 2018 Apr 19;13(4):e0195186. doi: 10.1371/journal.pone.0195186 (PMC5908175; doi:10.1371/journal.pone.0195186)
Supplement: S8 Table — CSF, cerebrospinal fluid; F, female; M, male; rhASA, recombinant human arylsulfatase A. (DOCX) [file pone.0195186.s009.docx]

**S8 Table.** **Individual and mean concentrations (ng/mL) of rhASA in serum and CSF in juvenile cynomolgus monkeys following intrathecal administration of rhASA 18.6 mg manufactured using process B.**

| **Source** | **Time point** | **Sex** | **Animal** | **Concentration (ng/mL)** |
| --- | --- | --- | --- | --- |
| Serum | Day 2 | M | 1 | 436 |
|  |  |  | 2 | 457 |
|  |  |  | 3 | 448 |
|  |  |  | 4 | 613 |
|  |  | F | 5 | 204 |
|  |  |  | 6 | 736 |
|  |  |  | 7 | 1030 |
|  |  |  | 8 | 424 |
|  |  |  | Mean | 544 |
|  | Week 11 | M | 1 | 49.2 |
|  |  |  | 2 | 0 |
|  |  |  | 3 | 0 |
|  |  |  | 4 | 114 |
|  |  | F | 5 | 0 |
|  |  |  | 6 | 0 |
|  |  |  | 7 | 0 |
|  |  |  | 8 | 126 |
|  |  |  | Mean | 36 |
| CSF | Day 2 | M | 1 | 681 |
|  |  |  | 2 | 1210 |
|  |  |  | 3 | 1000 |
|  |  |  | 4 | 1320 |
|  |  | F | 5 | 931 |
|  |  |  | 6 | 2220 |
|  |  |  | 7 | 9320 |
|  |  |  | 8 | 794 |
|  |  |  | Mean | 2185 |
|  | Week 11 | M | 1 | 318 |
|  |  |  | 2 | 126 |
|  |  |  | 3 | 185 |
|  |  |  | 4 | 495 |
|  |  | F | 5 | 27.9 |
|  |  |  | 6 | 81.5 |
|  |  |  | 7 | 75.1 |
|  |  |  | 8 | 59.0 |
|  |  |  | Mean | 171 |
|  |  |  |  |  |

CSF, cerebrospinal fluid; F, female; M, male; rhASA, recombinant human arylsulfatase A.
