# Supplementary material for: Nonclinical comparability studies of recombinant human arylsulfatase A addressing manufacturing process changes
Source: PLoS One. 2018 Apr 19;13(4):e0195186. doi: 10.1371/journal.pone.0195186 (PMC5908175; doi:10.1371/journal.pone.0195186)
Supplement: S9 Table — CSF, cerebrospinal fluid; F, female; M, male; rhASA, recombinant human arylsulfatase A. (DOCX) [file pone.0195186.s010.docx]

**S9 Table.** **Individual and mean concentrations (ng/mL) of anti-rhASA antibodies in serum and CSF in juvenile cynomolgus monkeys following intrathecal administration of rhASA 18.6 mg manufactured using process B.**

|  | **Sex** | **Animal** | **Concentration (ng/mL)** |  |
| --- | --- | --- | --- | --- |
| Serum | M | 1 | 61100 |  |
|  |  | 2 | 1110000 |  |
|  |  | 3 | 183000 |  |
|  |  | 4 | 5490 |  |
|  | F | 5 | 248000 |  |
|  |  | 6 | 846000 |  |
|  |  | 7 | 640000 |  |
|  |  | 8 | 38100 |  |
|  |  | Mean | 391461 |  |
| CSF | M | 1 | 1170 |  |
|  |  | 2 | 9190 |  |
|  |  | 3 | 3310 |  |
|  |  | 4 | 425 |  |
|  | F | 5 | 4700 |  |
|  |  | 6 | 21100 |  |
|  |  | 7 | 18200 |  |
|  |  | 8 | 962 |  |
|  |  | Mean | 7382 |  |

CSF, cerebrospinal fluid; F, female; M, male; rhASA, recombinant human arylsulfatase A.
